# Supplementary material for: Sensorimotor transformation underlying odor-modulated locomotion in walking Drosophila
Source: Nat Commun. 2023 Oct 26;14:6818. doi: 10.1038/s41467-023-42613-8 (PMC10603174; doi:10.1038/s41467-023-42613-8)
Supplement: Supplementary file 1 — Supplementary Information [file 41467_2023_42613_MOESM1_ESM.pdf]

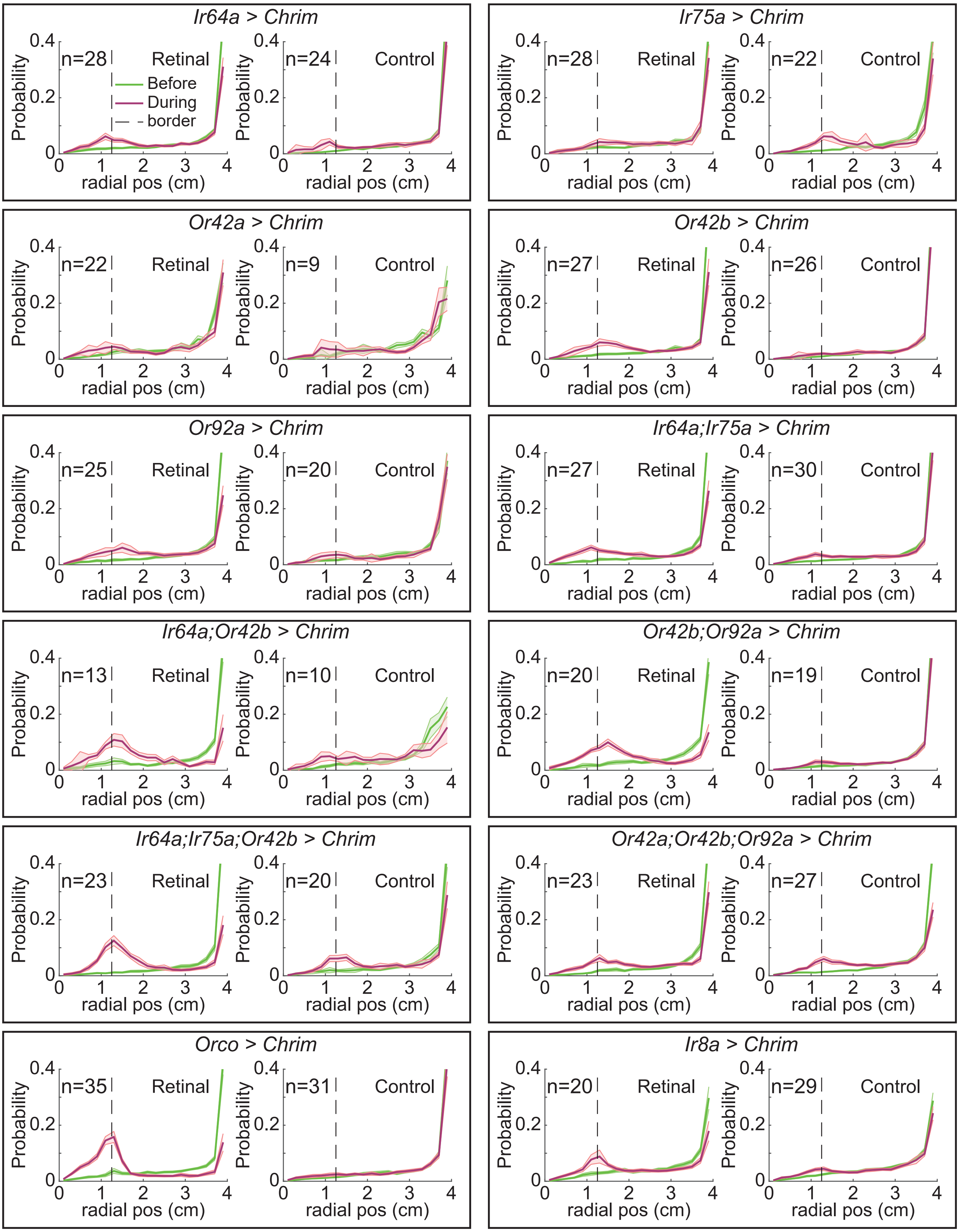

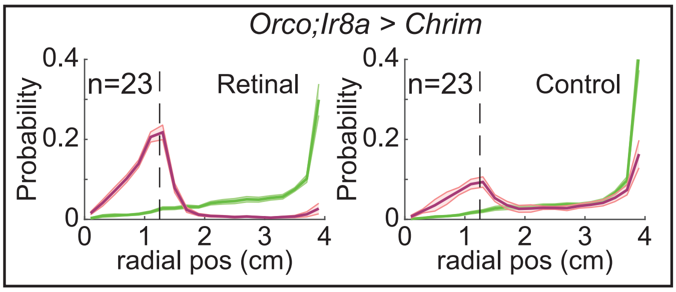

Figure S1. Radial occupancy of flies when different subsets of ORN classes are activated. Dotted lines show nominal light border (1.25 cm). Control flies were not fed retinal, which is necessary for optogenetic activation of neurons. Shaded region shows the SEM. Stops (speed<0.5 mm/s) are not considered here. n = individual animals.

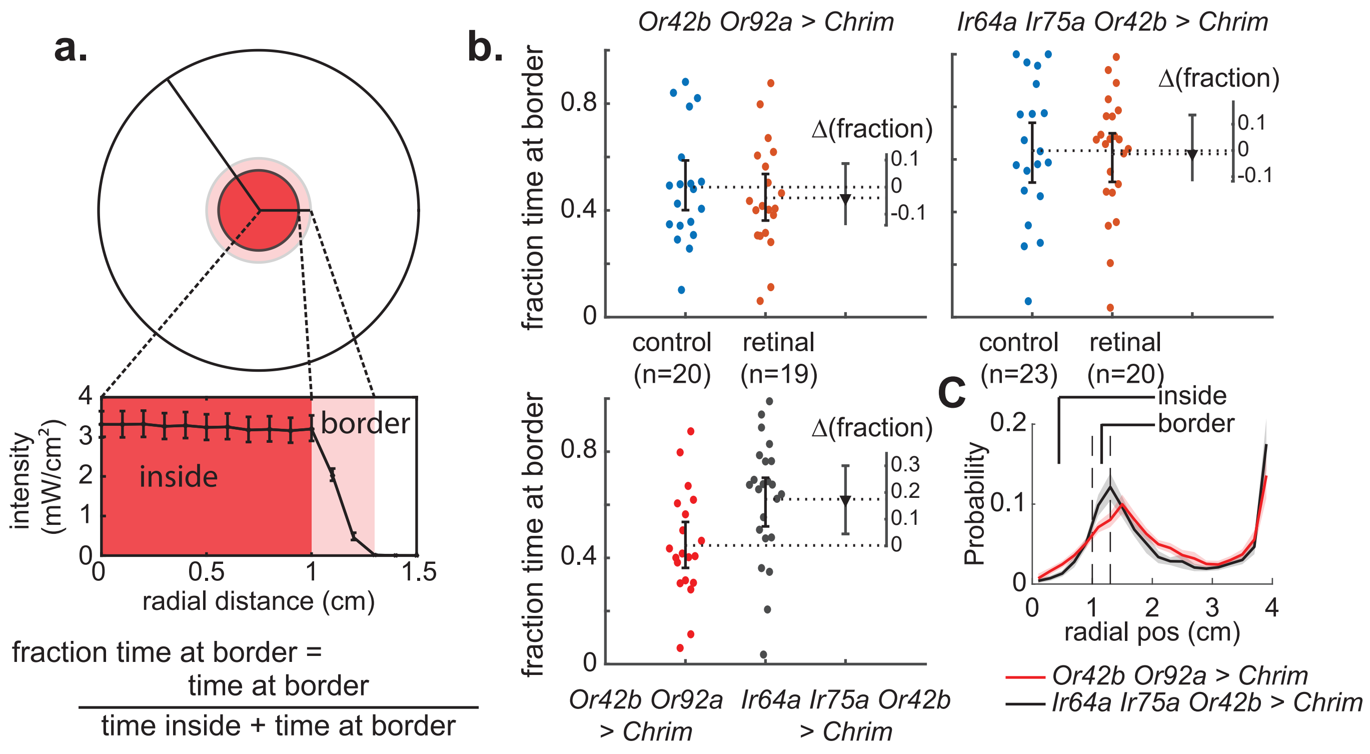

Figure S2. Flies whose *Ir64a Ir75a Or42b* are activated spend more time at the light border than do flies whose *Or42b Or92a* are activated. a. Schematic of calculation. The border is defined as between 1 and 1.3 cm from the center. We evaluate the fraction of time spent at the border **b.** The statistics plotted here use estimation graphics. Error bars show mean and bootstrapped 95% confidence intervals (see methods). Top: Proportion of time spent inside is not different between control and retinal flies for *Or42b Or92a > Chrim* and for *Ir64a Ir75a Or42b > Chrim*. Control flies are not fed retinal. Bottom: There is a difference in the proportion of time spent at the border between *Or42b Or92a > Chrim* and *Ir64a Ir75a Or42b > Chrim* flies fed on retinal. Based on the spatial area of each region, chance is 0.4083. **c.** This difference is reflected in the linear vs exponential like radial distribution of time flies inside and around the light zone.
n=individual animals

An interesting feature of these genotypes is that the control flies are scaled versions of retinal flies -

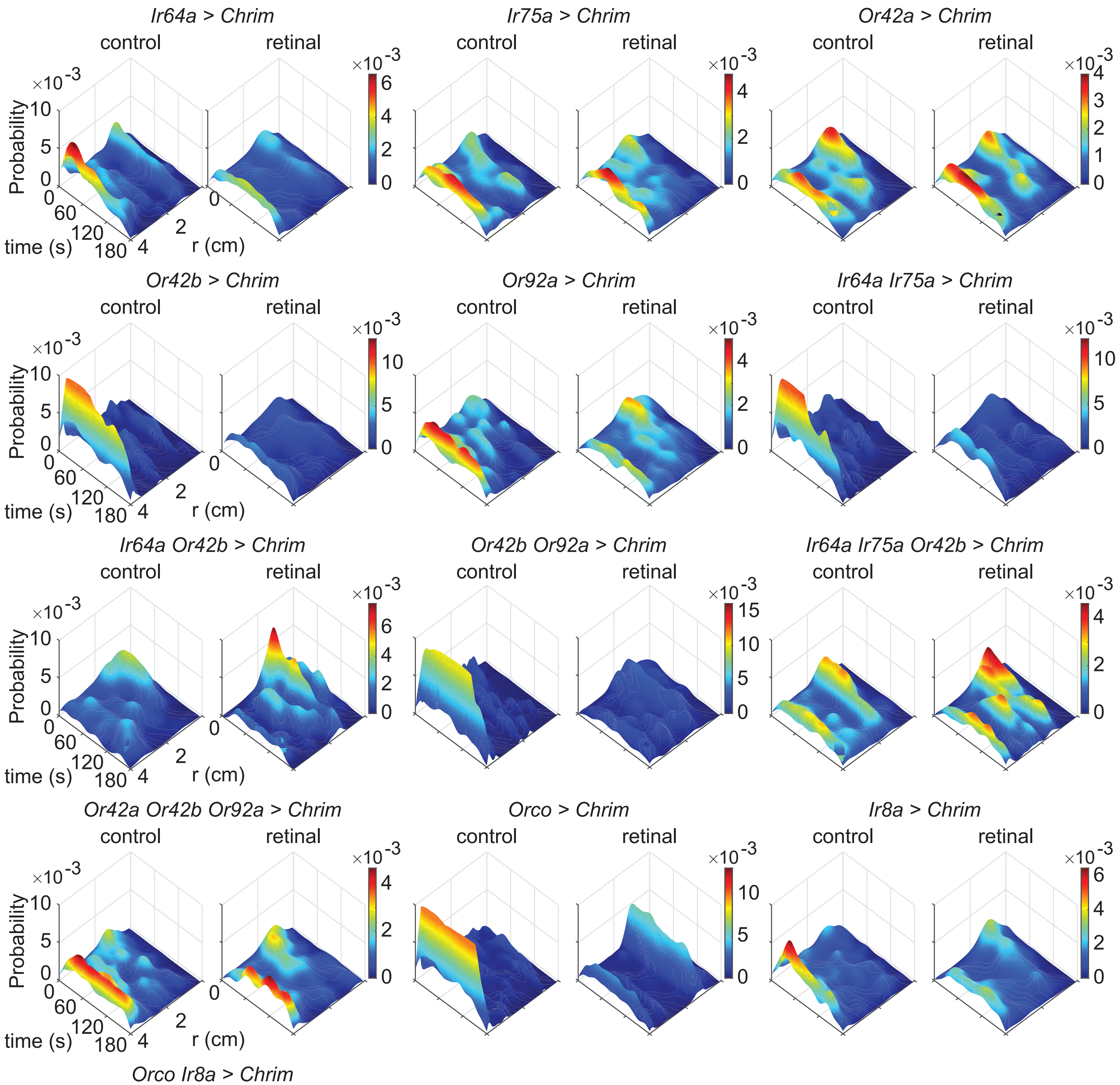

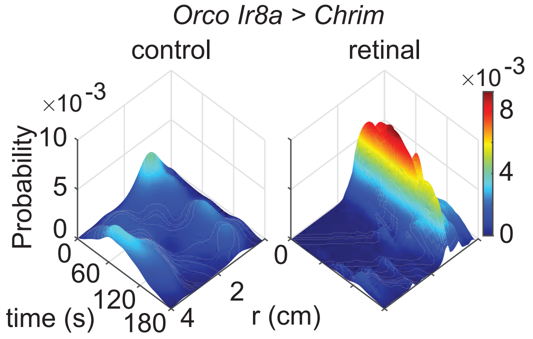

Figure S3. Spatio-temporal distribution of flies after first encounter with light for all retinal and control flies. The plots show the probability of finding the fly at a given radial distance from the center as a function of time. The color maps are paired by genotype but the Z-axis is kept the same across genotypes.

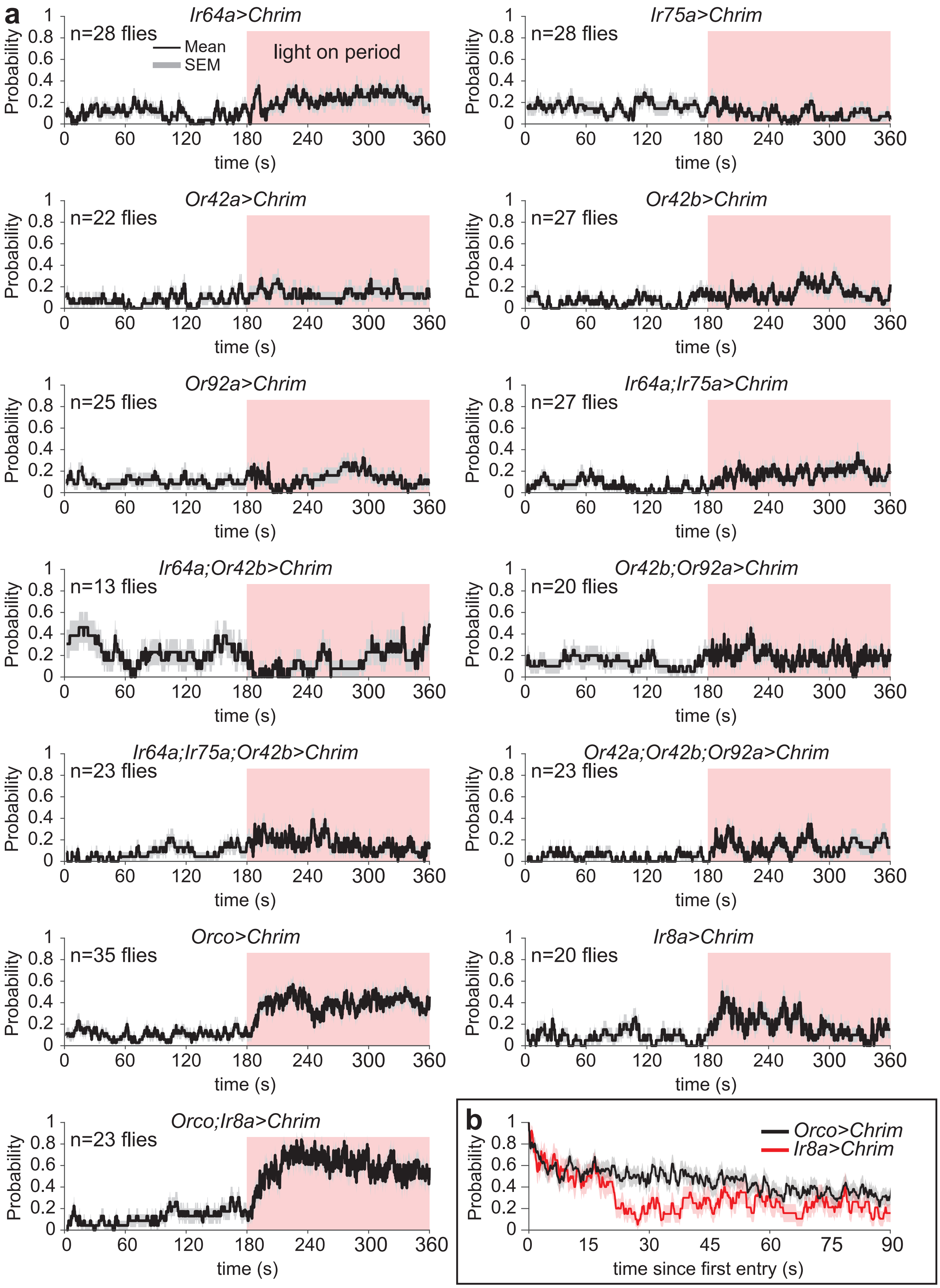

Figure S4. Proportion (probability) of flies located in the light zone under optogenetic stimulation of different subsets of ORN classes. a. The nominal light border is 1.25 cm. Light turns on at the 3 minute mark (180 seconds). **b.** Proportion of flies in the light zone after aligning flies by first entry after the 3 minute mark for *Orco>Chrim* and *Ir8a>Chrim*. There is a massive drop in the proportion of *Ir8a* flies inside the light zone around the 17 second mark. Plots show mean +/- SEM.

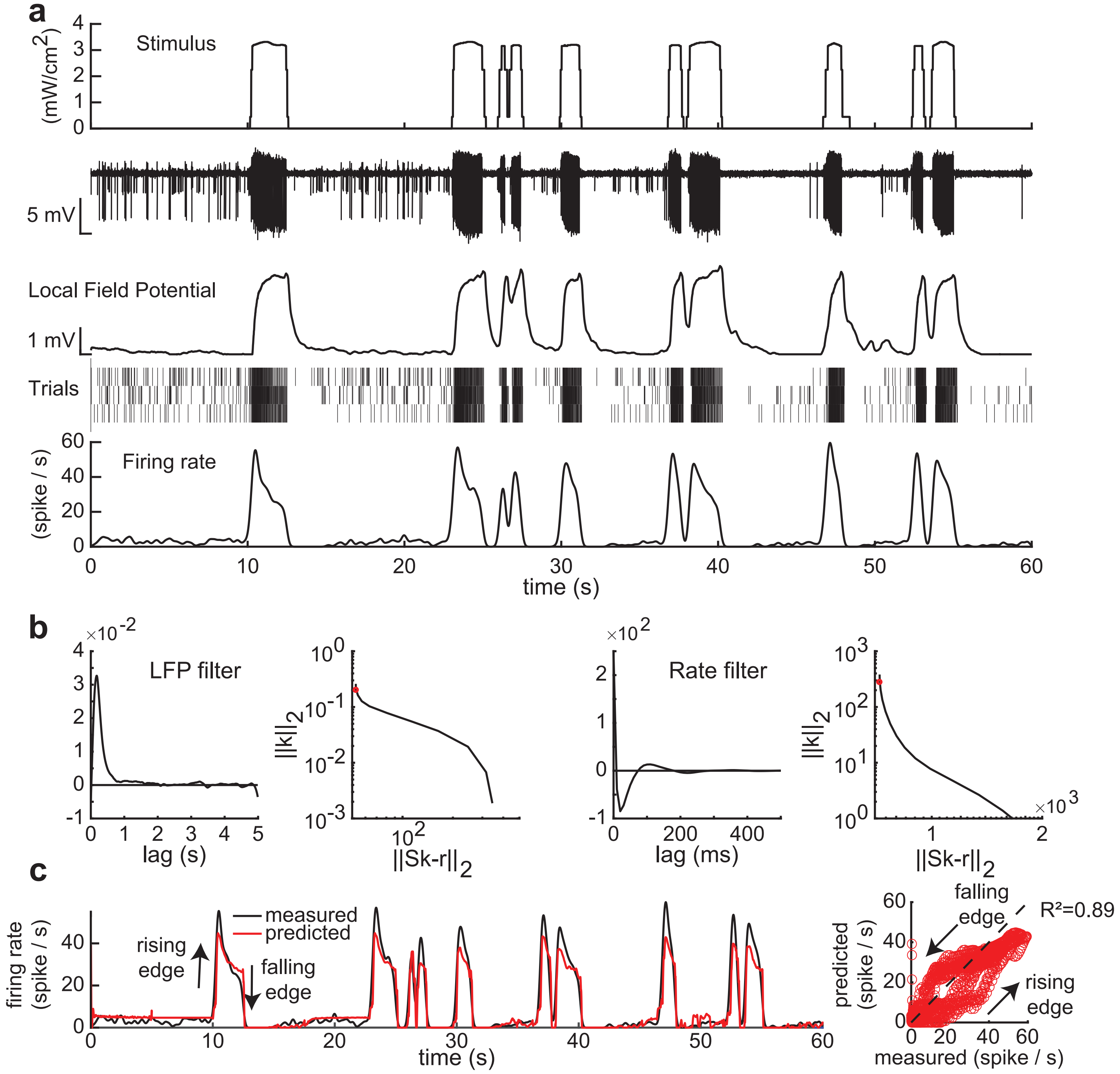

Figure S5. ORN responses, construction and validation of encoder . a. From the top to the bottom: A sample 60 second light stimulus; local field potential subtracted trace to show spikes; median filtered trace showing local field potential; Raster plot of 3 sensilla recordings for the stimulus pattern; the average firing rate profile over the 3 trials was estimated using a 150 ms gaussian kernel. **b. (leftmost panel)** A linear filter was calculated using Tikhonov regularization ($\|Sk-r\|^2+\|\lambda Ik\|^2$) to transform from the stimulus to the local field potential. **b (second from the left).** The H-curve with the regularization parameter (red dot) corresponding to the LFP filter. (**Right two panels)** same as the left two panels but for the linear filter transformation from the local field potential to the firing rate. **c. Left.** Measured (black) and filter predicted firing rates (red dot) for this stimulus pattern. The filter predictions underestimate the peak responses. **Right.** Plot of the measured and predicted against each other. The dotted line is the identity line. In general, the filter predictions is highly correlated to the measured, but underestimate the rising edge and overestimate the falling edge.

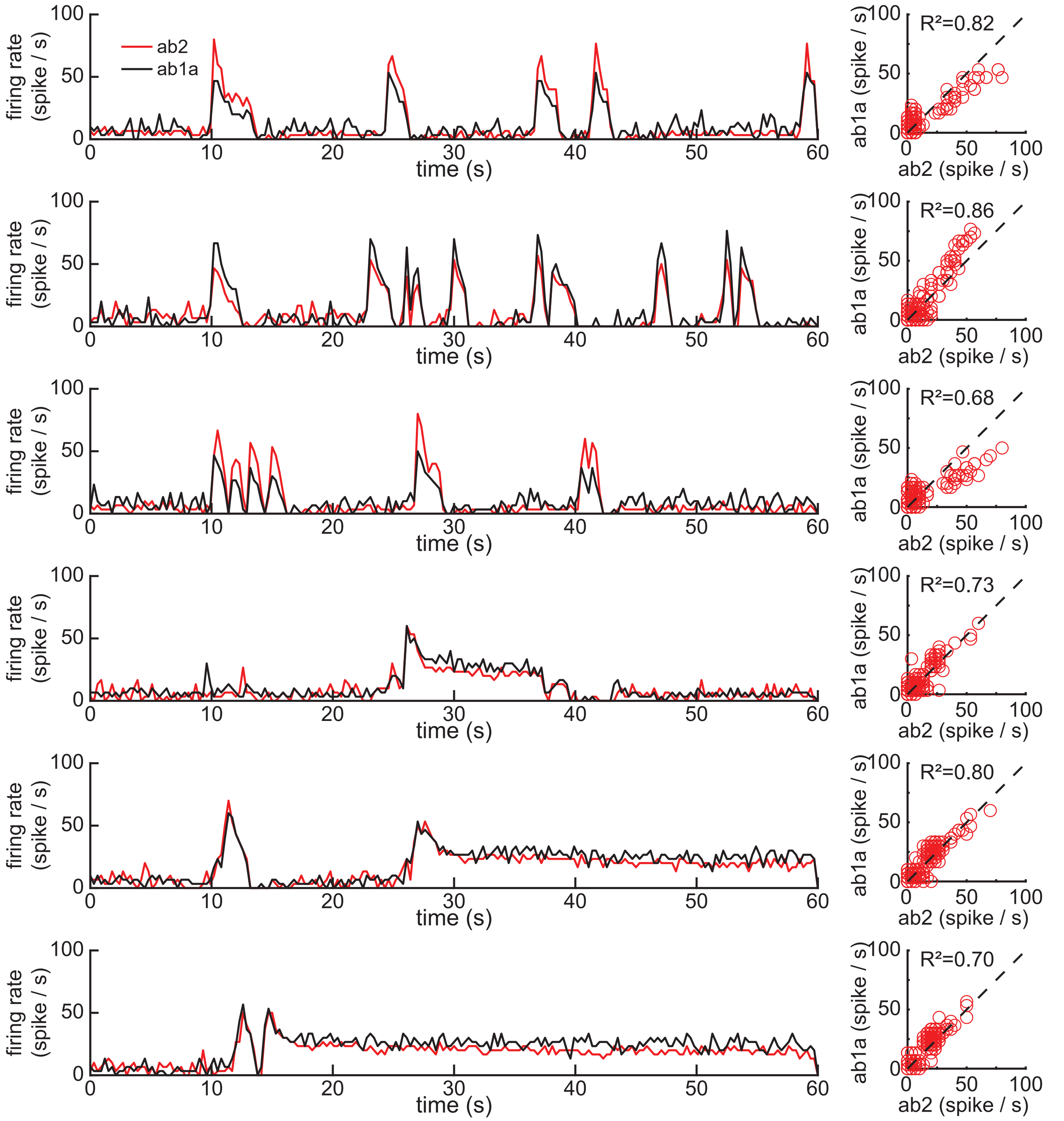

Figure S6. Optogenetic activation of ORNs trigger similar firing rate responses across ab1a and ab2 sensilla. Left. Each row shows firing histogram for a single trial of recordings from ab2 and ab1a sensilla in response to six types of stimulus pattern. **Right.** Scatter plot of the ab2 and ab1a histogram traces against each other. The dotted line is the identity line.

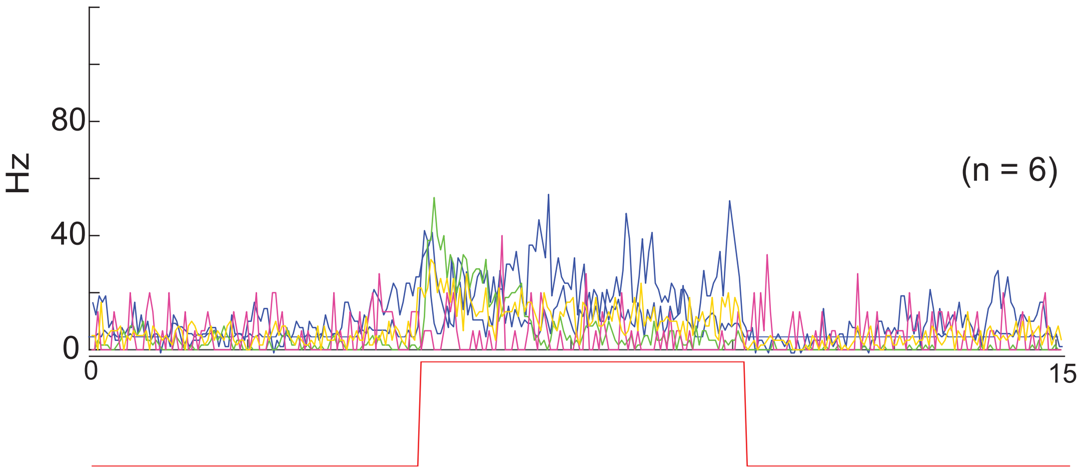

Figure S7. Control experiments suggest that there is a small response in the control flies. Preliminary recordings suggested that the ORN responses in control flies are small if at all present. To evaluate control responses with more certainty, we recorded from the downstream PN in flies where Orco-Gal4 drives the expression of UAS-Chrimson. We recorded from PNs because weak responses in ORNs are amplified in the PNs. PSTHs from PNs (each trace is an average of 6 trials) show that some PNs did respond to light stimulation. These responses are small compared to the responses observed even in the ORNs in the presence of retinal.

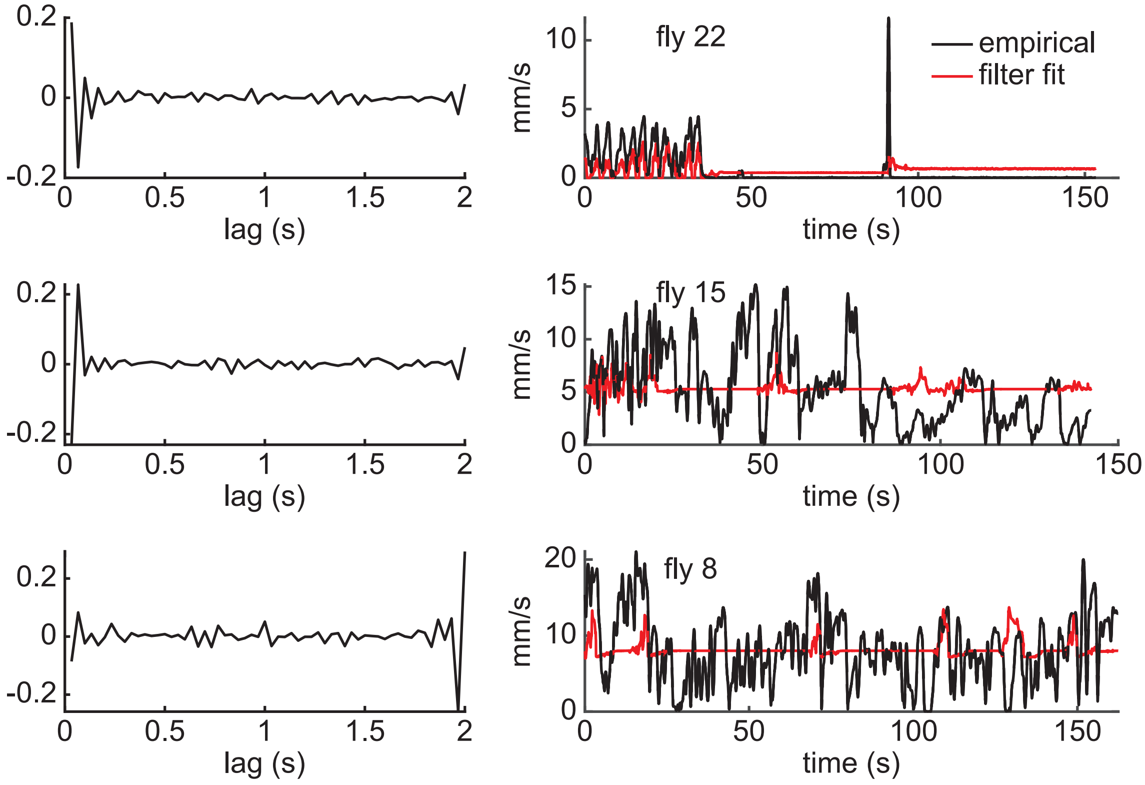

Figure S8. Fly specific linear filter predictions of movement speed for *Orco* flies do not match empirical data. We attemped to obtain a linear filter that describes the relationship between firing rate and speed. The top row shows a fly where the filter shows temporal structure and the predicted speed generally follows the fly's speed. Middle row shows a case where the filter again shows temporal structure, but the predicted speed matches poorly to actual movement speed. Bottom row shows a case where the filter shows poor temporal structure (peak at 2 seconds) and the predicted speed matches poorly to actual movement speed.

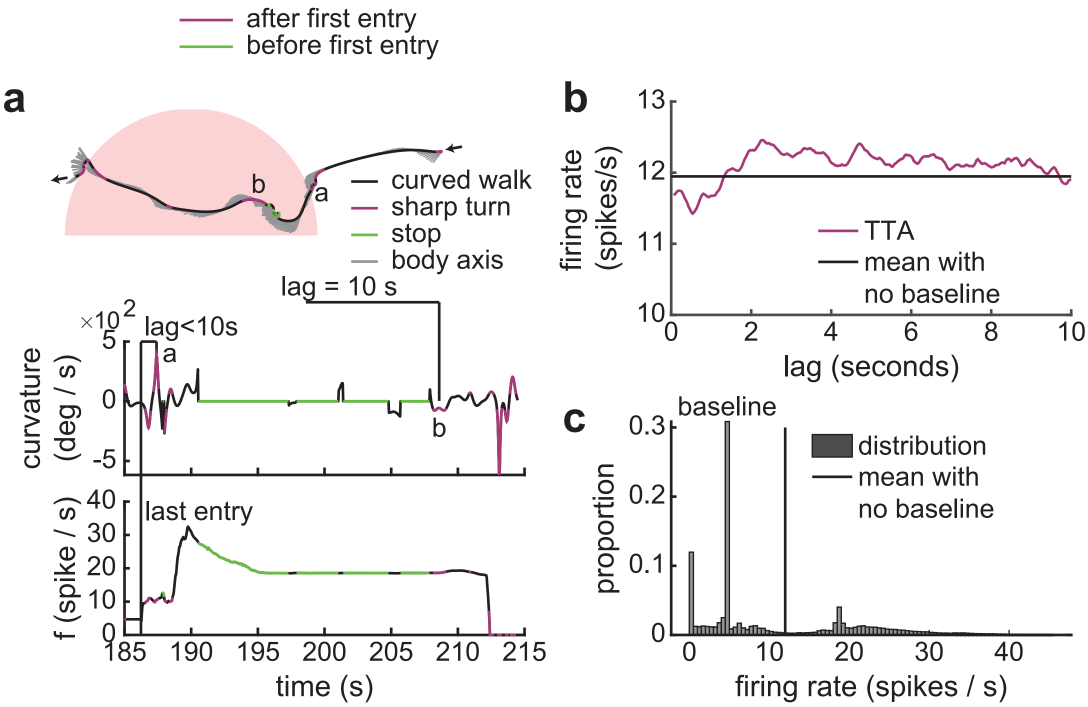

Figure S9. Turn triggered average as an example of reverse-correlation approach. The approach works qualitatively but has limitations. a. Schematic illustrating how turn triggered average (TTA) was estimated. **Top:** A sample trajectory from a *Orco>Chrimson* fly. The fly's body path is shown in black with sharp turn segments in red and stop segments in green. The fly's body axis to the head is shown as gray segments. Pink shaded semicircle indicates light zone. The two arrows indicate the direction of movement. Two sample sharp turns are labeled as a and b. **Middle:** The curvature of the body path is shown for this segment. The peak curvature marked the time of the sharp turn. The turn triggered firing rate for each sharp turn was defined as the firing rate (**bottom**) from 10s prior to the sharp turn i or up to the last entry if the turn occurs within 10s of last entry into the stimulated region. The turn triggered average (TTA) is defined as the mean of the turn triggered firing rate for all sharp turns. The curvature during stops were set to 0 for visualization because curvature becomes noisy and blows up as speed goes to zero. **b.** The turn triggered average (TTA). As expected a drop in the firing preceeds the turn. However, the mean does not return to zero and the breadth of the negative peak is long. These features of the TTA reflect stimulus characteristics. **c.** The stimulus (spike-rate) is highly non-Gaussian invalidating the TTA.

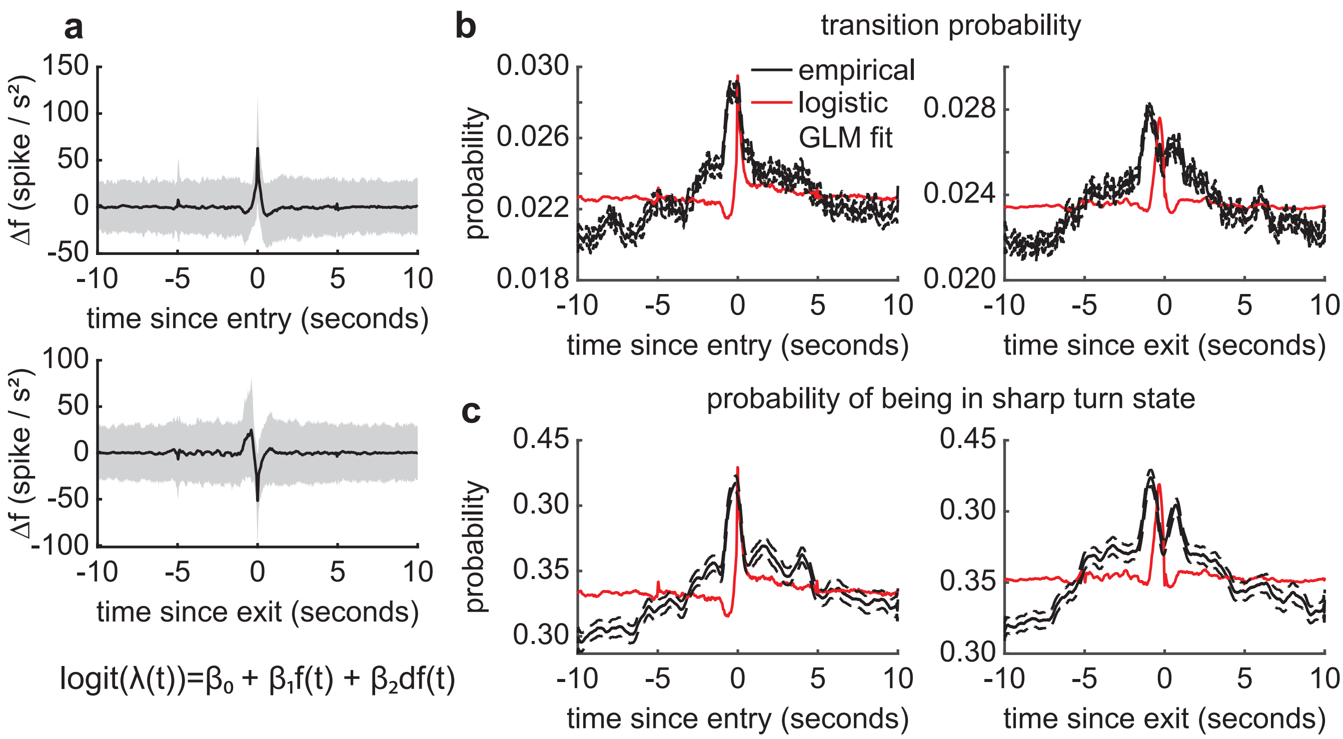

Figure S10. Generalized linear model for the probability of turning after entering and leaving the light zone for *Orco>Chrimson* does not fit the experimental data. a. Fly tracks are aligned by a peak in the change in firing rate for entry and by the negative peak in firing rate for leaving. A GLM was fit to the probability of turning using the instantaneous firing rate and the instantaneous change in firing rate. Error bars show standard deviation (n=2178 entrys and 2700 exits). **b.** GLM for the probability of transitioning into a sharp turn. Empirical turn location is based on when a curved walk transitioned into a sharp turn state. The model is a poor fit to data. **c.** GLM for the probability of being in a sharp turn. Here, probability is defined as the proportion of flies in a sharp turn state. Error bars for empirical probabilities is standard deviation based on bootstrap sampling of crossings. As in B, the model is a poor fit to data.

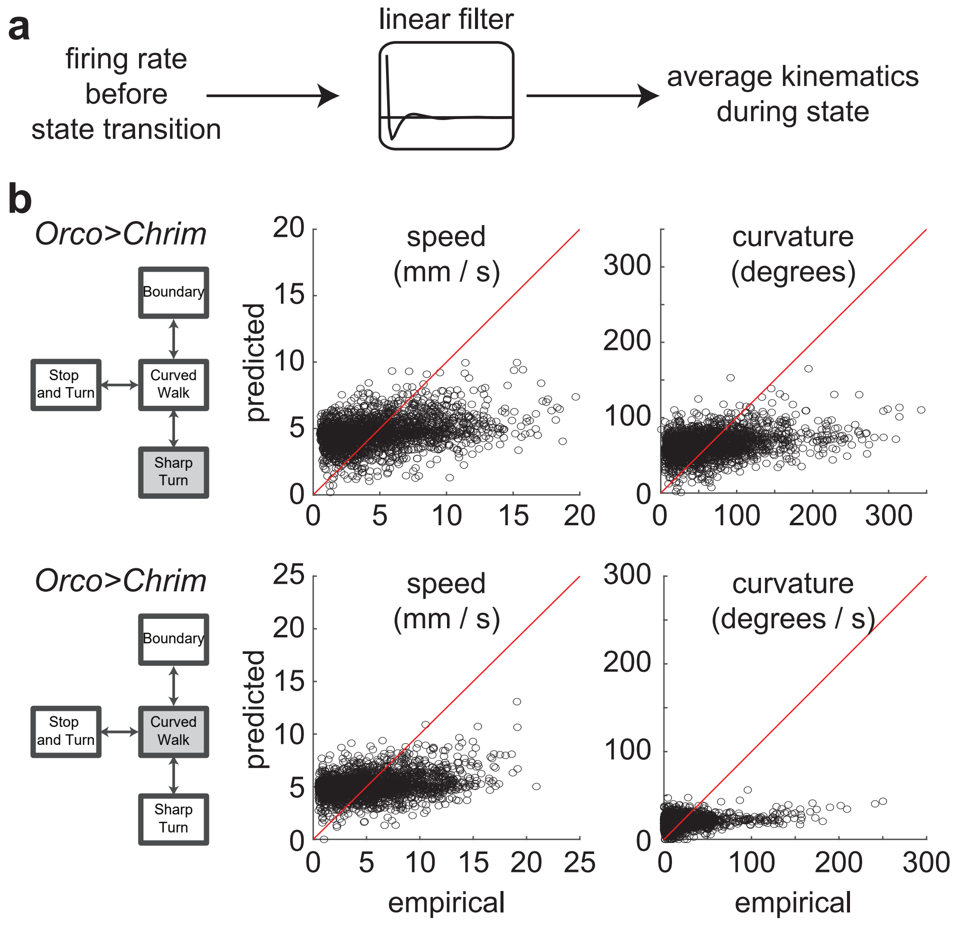

Figure S11. Linear filter predictions for time averaged kinematics during each sharp turn and curved walk state trajectory. a. Schematic illustrating analysis. A continuous time linear filter is convolved with the firing rate prior to each state transition to predict the average speed and curvature during the next state trajectory. **b.** Average speed and curvature predictions from the linear filter plotted against the empirical values. Top row corresponds to sharp turn and bottom row corresponds to curved walk. The line of unity is shown in red.

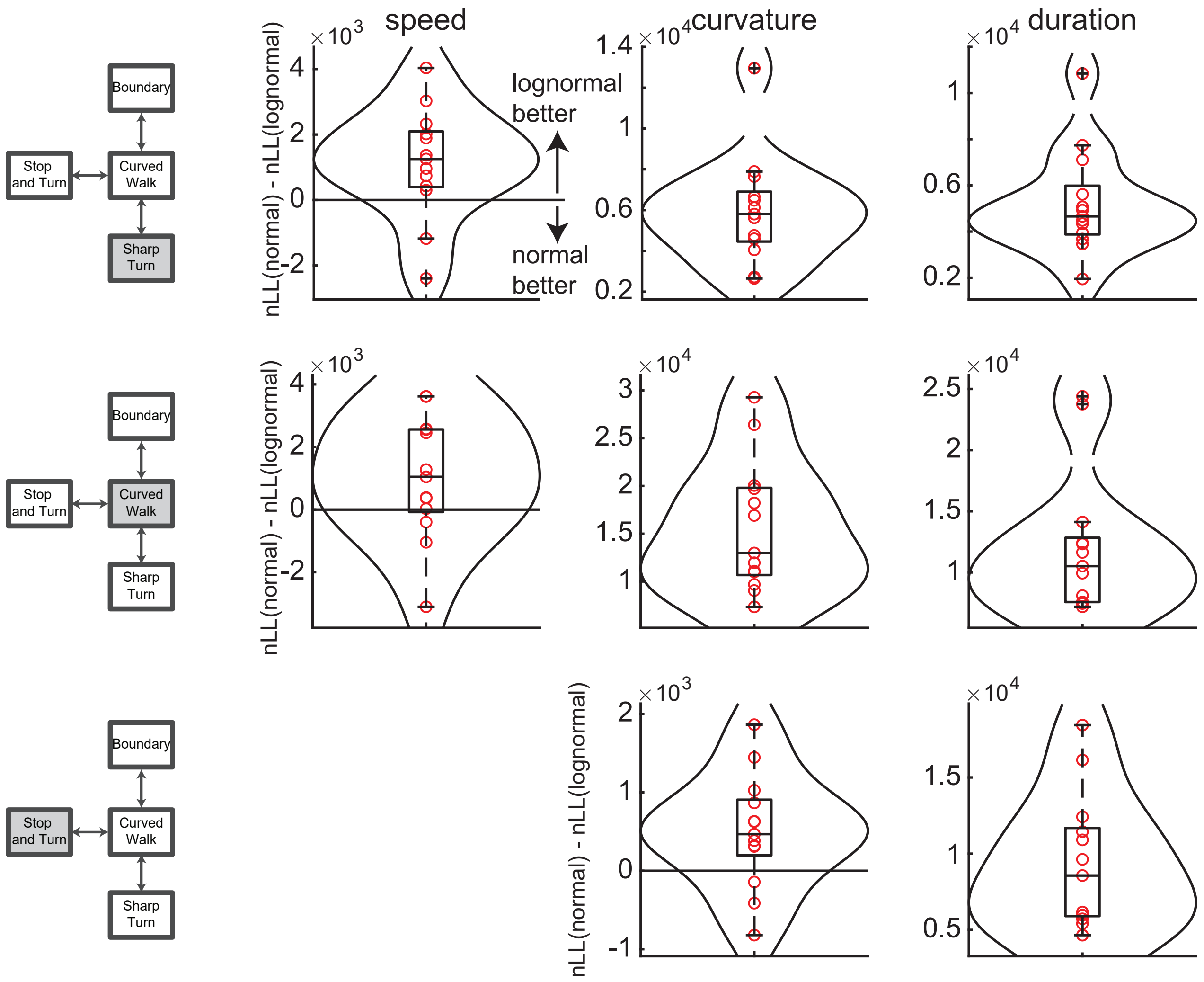

Figure S12. KNN samples fit better to lognormal distributions than normal distributions. Violin plots show the kernel distribution of the difference in negative log-likelihood (nLL) fits for KNN samples used to estimate the (f, df) space for state kinematics. Each dot corresponds to the difference in nLL fit of each ORN genotype. Box and whisker plots show the ourlier removed minimum, 25th percentile, median, 75th percentile, and max values. A positive difference indicates that the data is better fit with lognormal than normal distributions. A zero line is draw for state kinematics where the distribution of difference in nLL crosses 0. In all there are 130 distributions, and lognormal is better for all but 8 of them.

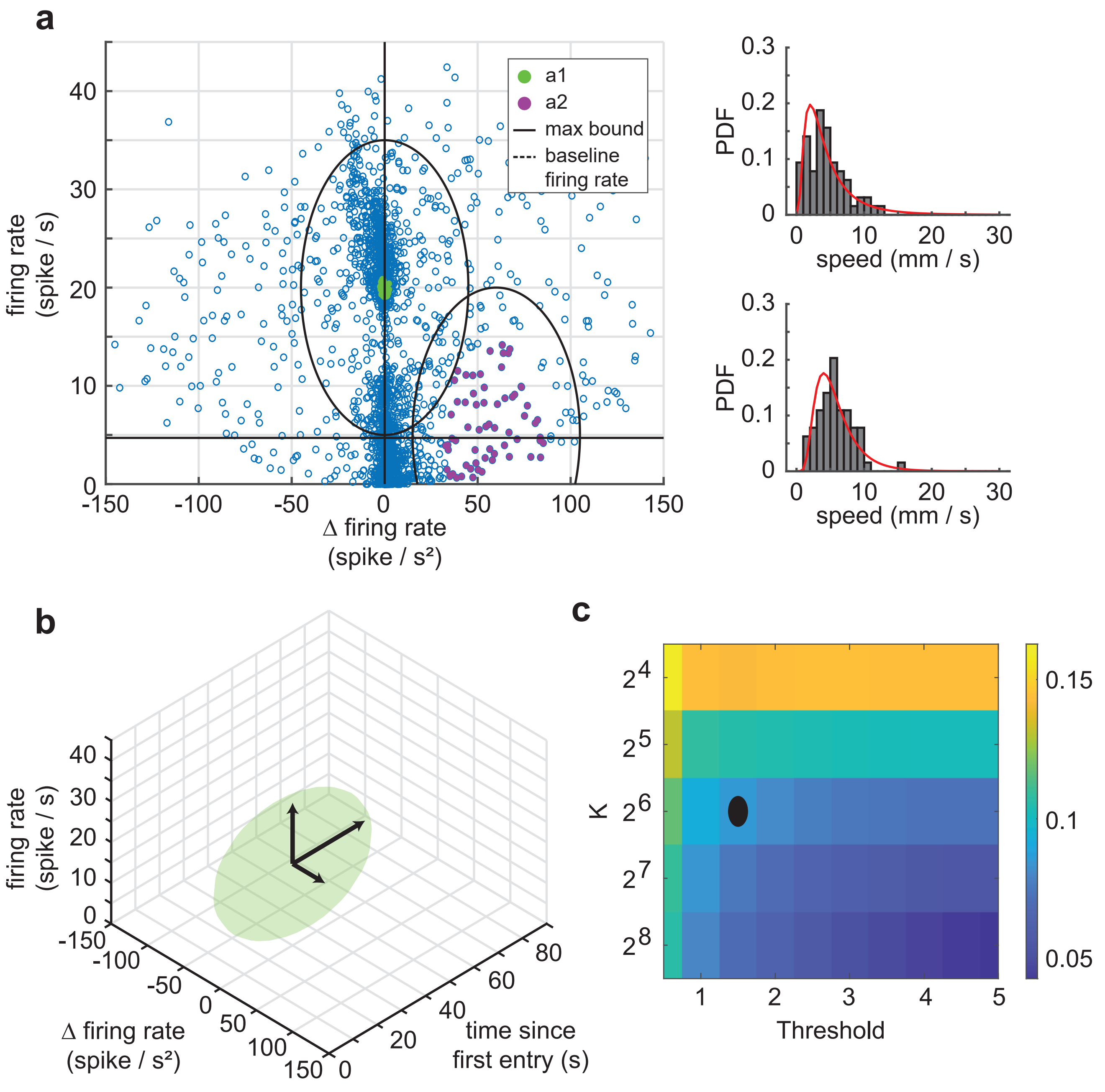

Figure S13. K-nearest neighbor (KNN) approach to estimating how ORN activity drives locomotor kinematics. KNN approach for the estimation of speed of the curved walk. **a.** Each blue point is the average firing rate and change in firing rate of ORNs 200 ms prior to the start of a curved walk. Therefore, each blue point is associated with a curved walk that is just about to start and therefore a single speed value reflecting the averge speed of that curved walk. At each location in this grid, the K nearest trajectories are used to estimate a log-normal probability density function (PDF). The probability density functions (right two plots) shows example log-normal PDF fits of curved walk speed corresponding to the green dots around 20 spikes/s firing rate, 0 spikes/s² change in firing rate and red dots around 5 spikes/s firing rate, 60 spikes/s² change in firing rate respectively. Dotted black line shows the baseline firing rate. Black solid line indicates the set maximum bounding distance. **b.** The maximum bound of the pdf in **a** extended to account for changes in ORN effect on locomotion due to adaptation. **c.** K and the maximum bounding oval threshold is determined by finding the inflection point (black dot) in minimizing the standard error of the mean across all grid points in the 3D space.

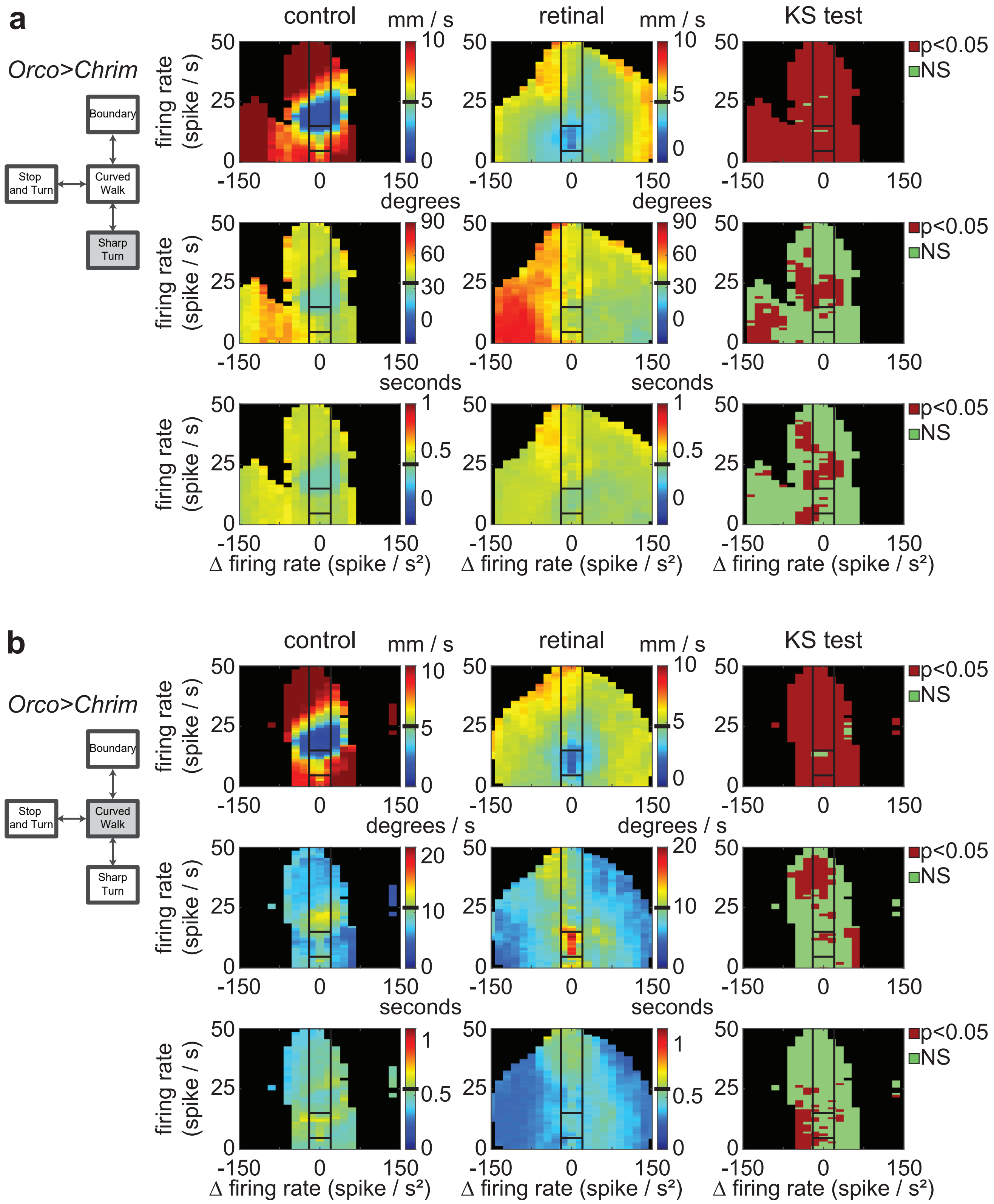

Figure S14. Differences in KNN estimation of locomotor parameters between *Orco>Chrim* flies not fed retinal (control) and those fed on retinal. a. Sharp turn speed, curvature, and duration. Significant changes (Kolmogorov–Smirnov test, $p<0.05$) in the KNN space are shown in red in the KS test image. Since ORNs in control flies should not respond to optogenetic stimulation, the KNN space for control flies were computed from fictious firing rate traces where the light stimulus experience over time is convolved with the linear filters generated from retinal flies. **b.** Same as A, but for curved walks.

Note: control data is presented for completeness but it is hard to interpret as control flies cross through the central region rarely. Much of the little data corresponds to times when they are already inside and almost stopped or just quickly walking through accounting for really large and small speeds.

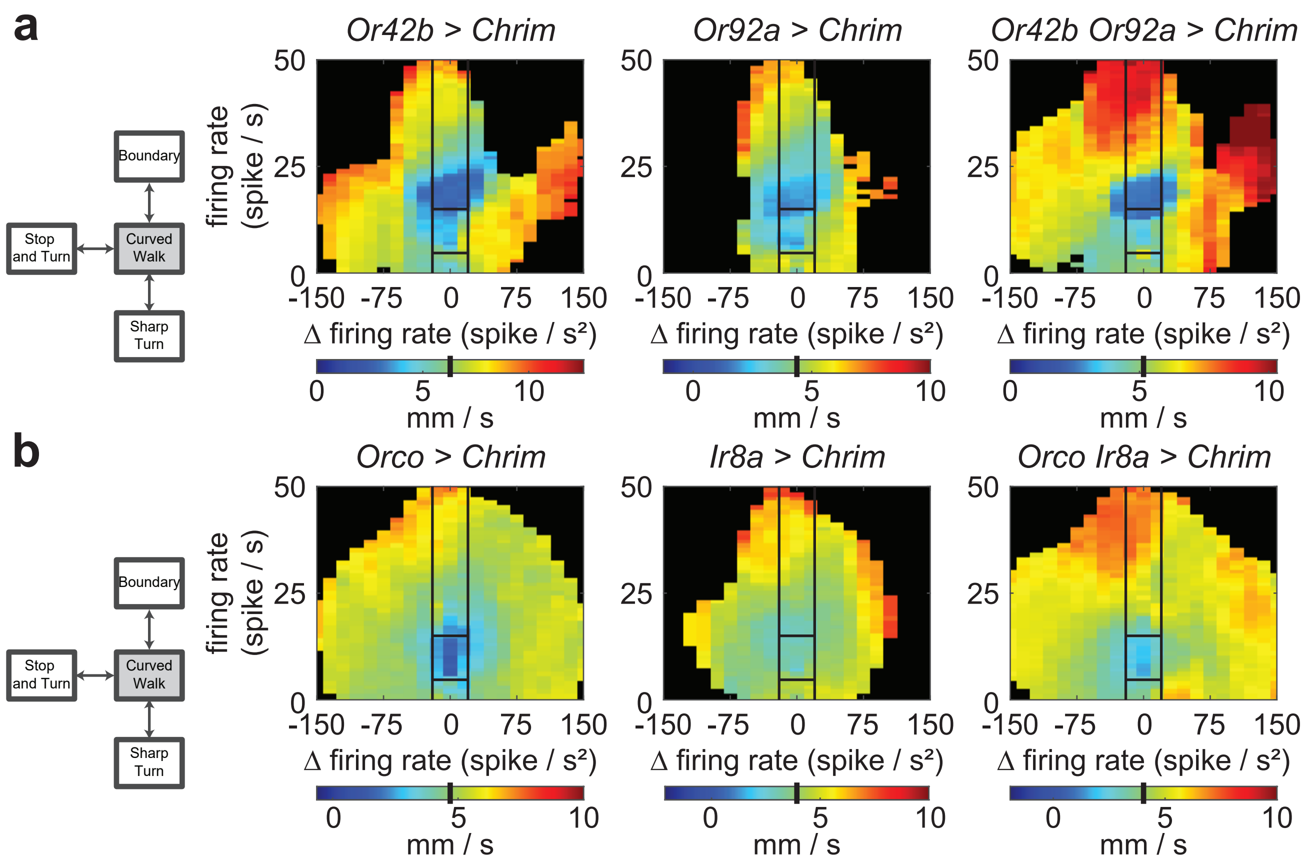

Figure S15. Some locomotor parameters are more affected when a small number of ORN classes are active. a. Curved walk speed is strongly affected by activation of a single ORN class (*Or42b* and *Or92a*). The effect is even larger when the two are activated together. Black lines separate out the sensorimotor mapping into 5 broad regions based on firing rate and change in firing rate. Baseline speed is shown as a black bar in the colormap. **b.** The effect of activated large populations of neurons that labels the co-receptor *Orco*, *Ir8a*, or either is smaller than that of individual ORNs.

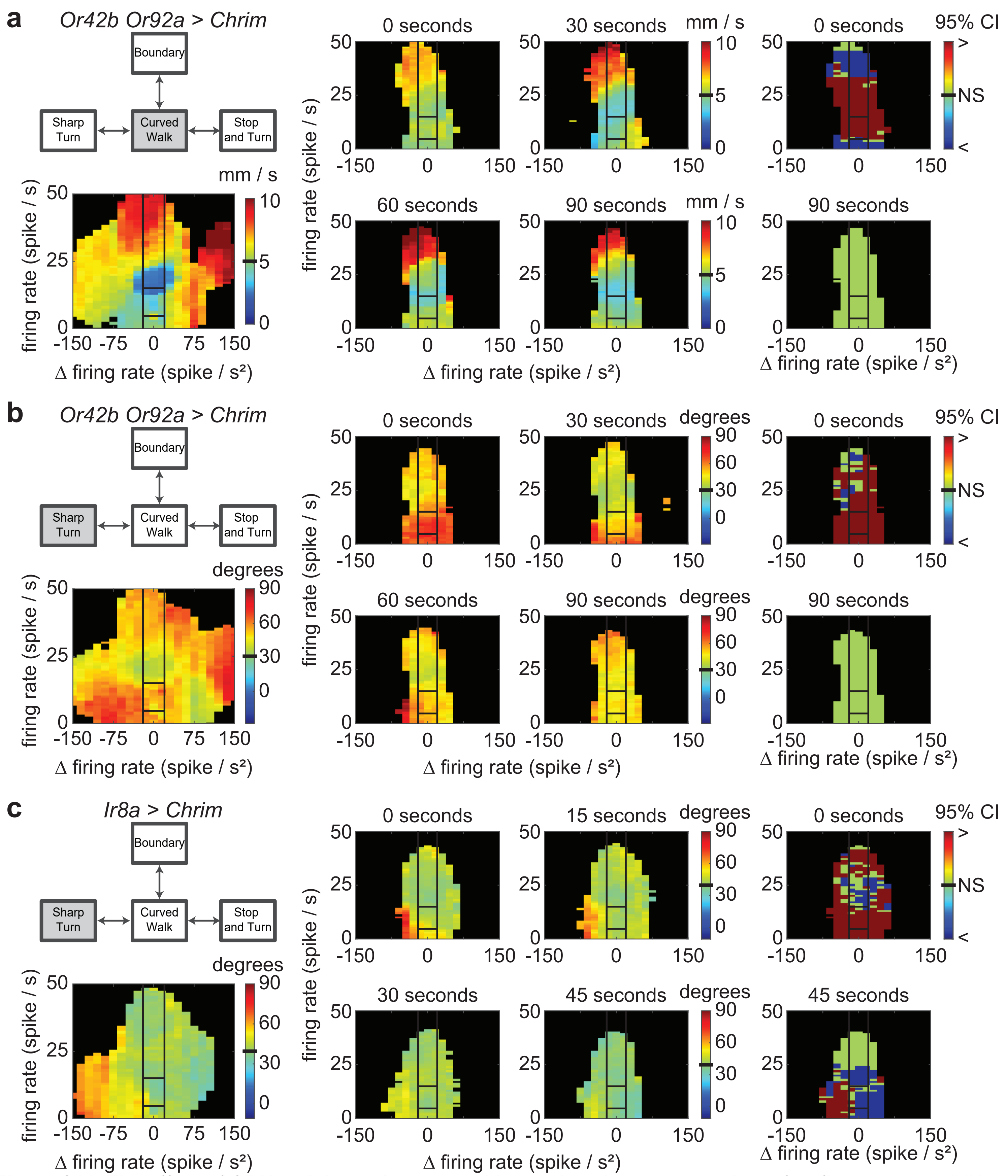

Figure S16. The effect of ORN activity on locomotor kinematics changes over time after first entry. a. KNN estimate of curved walk average speed for *Or42b* and *Or92a* > Chrim across all time (**large color map on left**). KNN mapping of ORN activity to curved walk speed shows a large increase in speed within the first 30 seconds since first entry that does not continue to adapt. Pemutation test (extreme right) shows how different parts of the KNN space relax to time shuffled estimations of the KNN space. **b.** KNN estimate of sharp turn curvature for *Or42b* and *Or92a* > Chrim across all time (**large color map on left)**. KNN mapping of ORN activity to sharp turn curvature showing the higher curvature at high firing rates and during inhibition adapts, but is still present after 90 seconds since first entry. **c.** KNN estimate of sharp turn curvature for *Ir8a* > Chrim across all time ((**large color map on left**).). KNN mapping of ORN activity to sharp turn total curvature showing the higher curvature due to a sharp drop in ORN activity adapts over time.

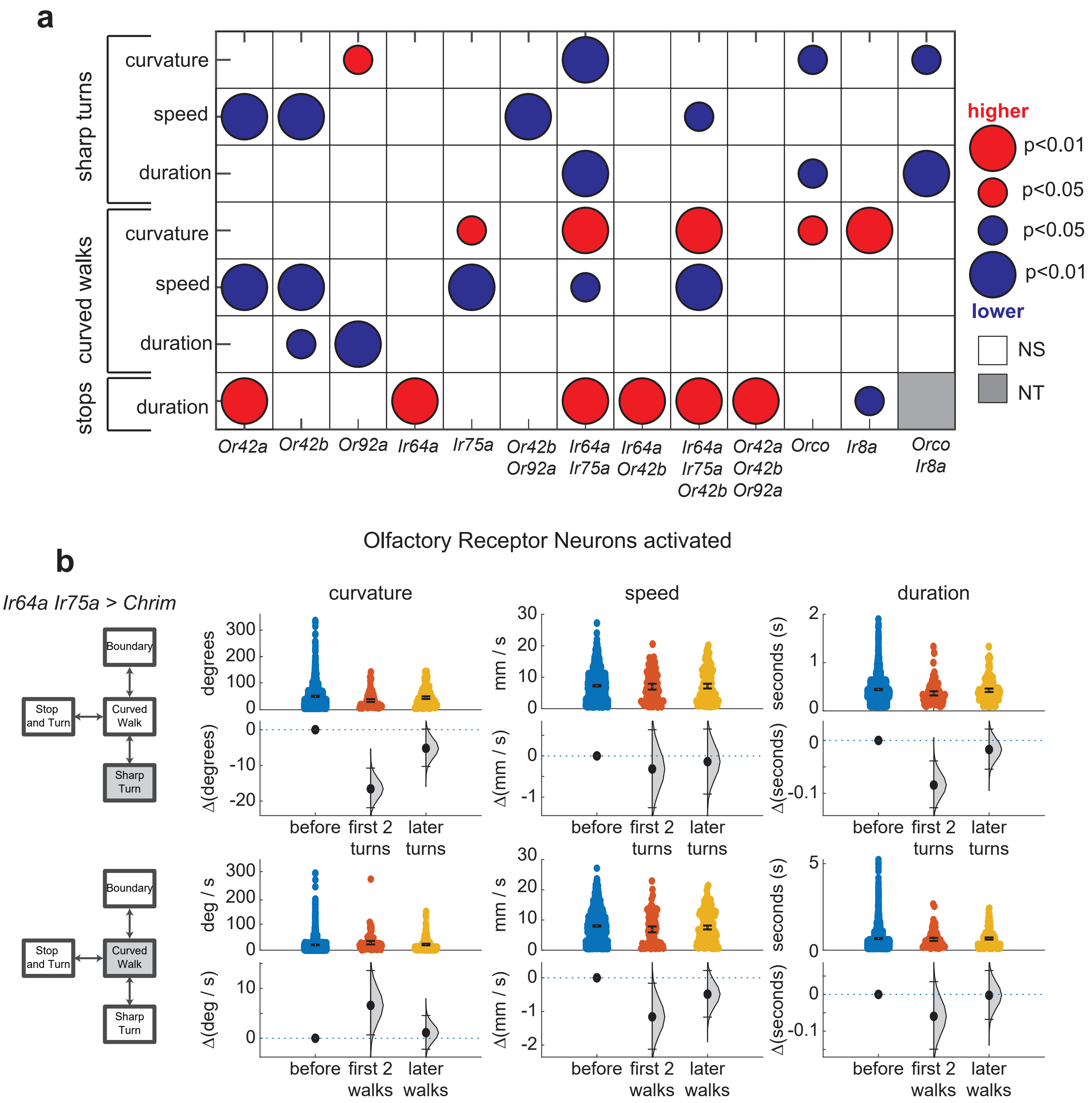

Figure S17. Previous ORN experience has limited effect on locomotion after ORN activity reaches baseline. a. One sided Wilcoxon rank sum tests comparing the sharp turn, curved walk kinematics and stop duration when the fly loses the odor source (ORN activity reaches baseline firing rate) is higher than (red) or lower than (blue) baseline values before first entering the light ring. Circle size shows significance at 0.05 and 0.01. Parameters that were not significant (NS) are in white. Parameters that had less than 30 samples were not tested (NT). **b.** The statistics plotted here use estimation graphics. Bootstrapped distribution of difference with 95% confidence marked is plotted (see methods). For flies where ORN experience does have an effect on baseline kinematics, this kinematics adapts over time. *Ir64a* and *Ir75a* > Chrim retinal flies perform less curved sharp turns for lower time durations after recently reaching baseline firing. After spending more time at baseline, the sharp turn curvature and duration adapts to baseline levels observed before first entry. (n for curved walk = 2099 before, 110 first 2 and 231 later; n for sharp turn = 1560 before, 101 first 2

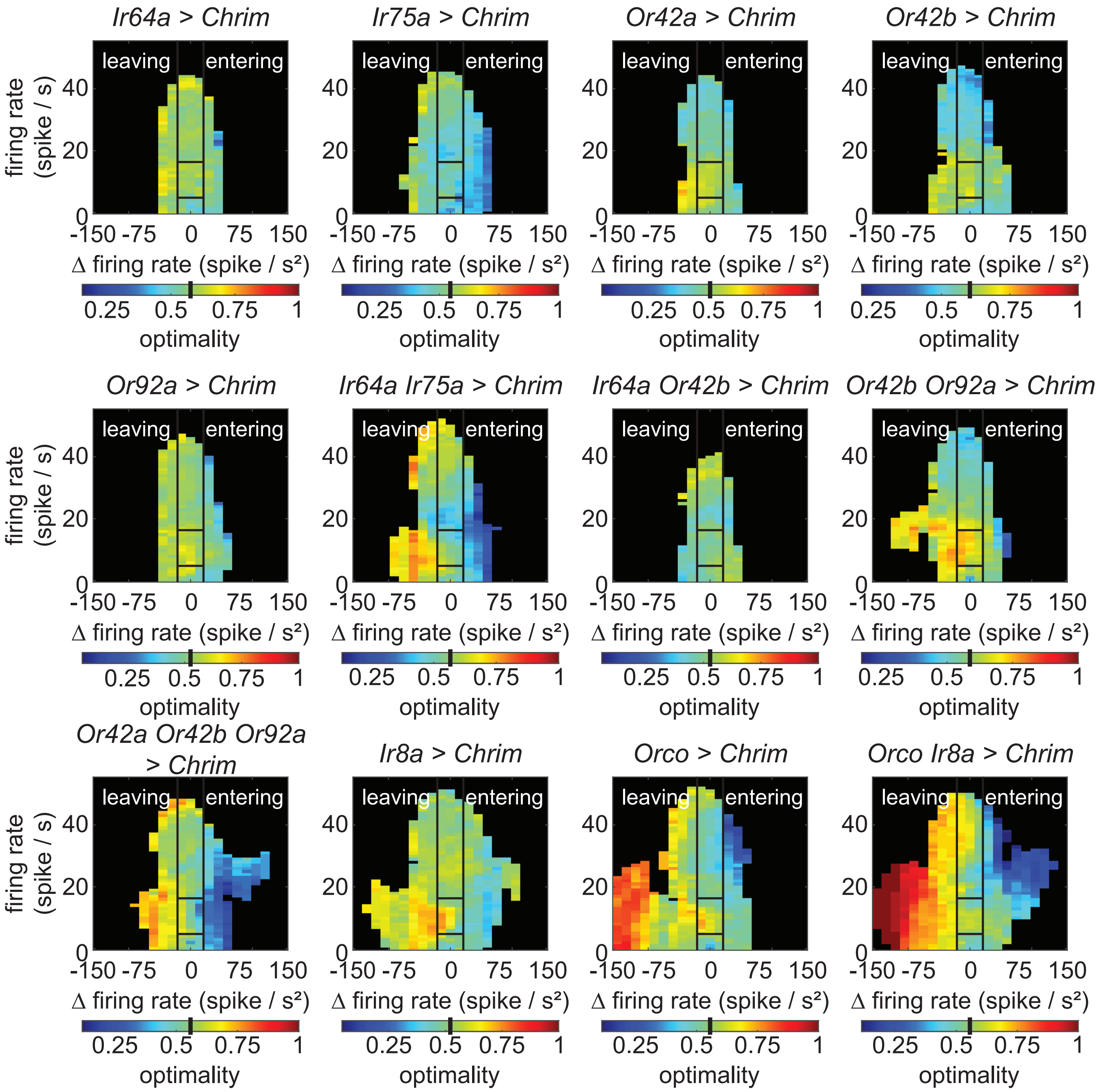

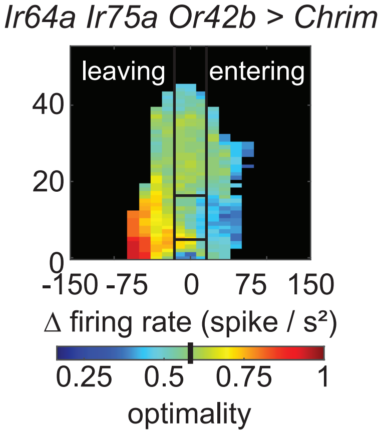

Figure S18. Turn optimality for control flies. Turn optimality (defined as the percentage of optimal turns) for all combinations of ORN classes where flies are not fed retinal.

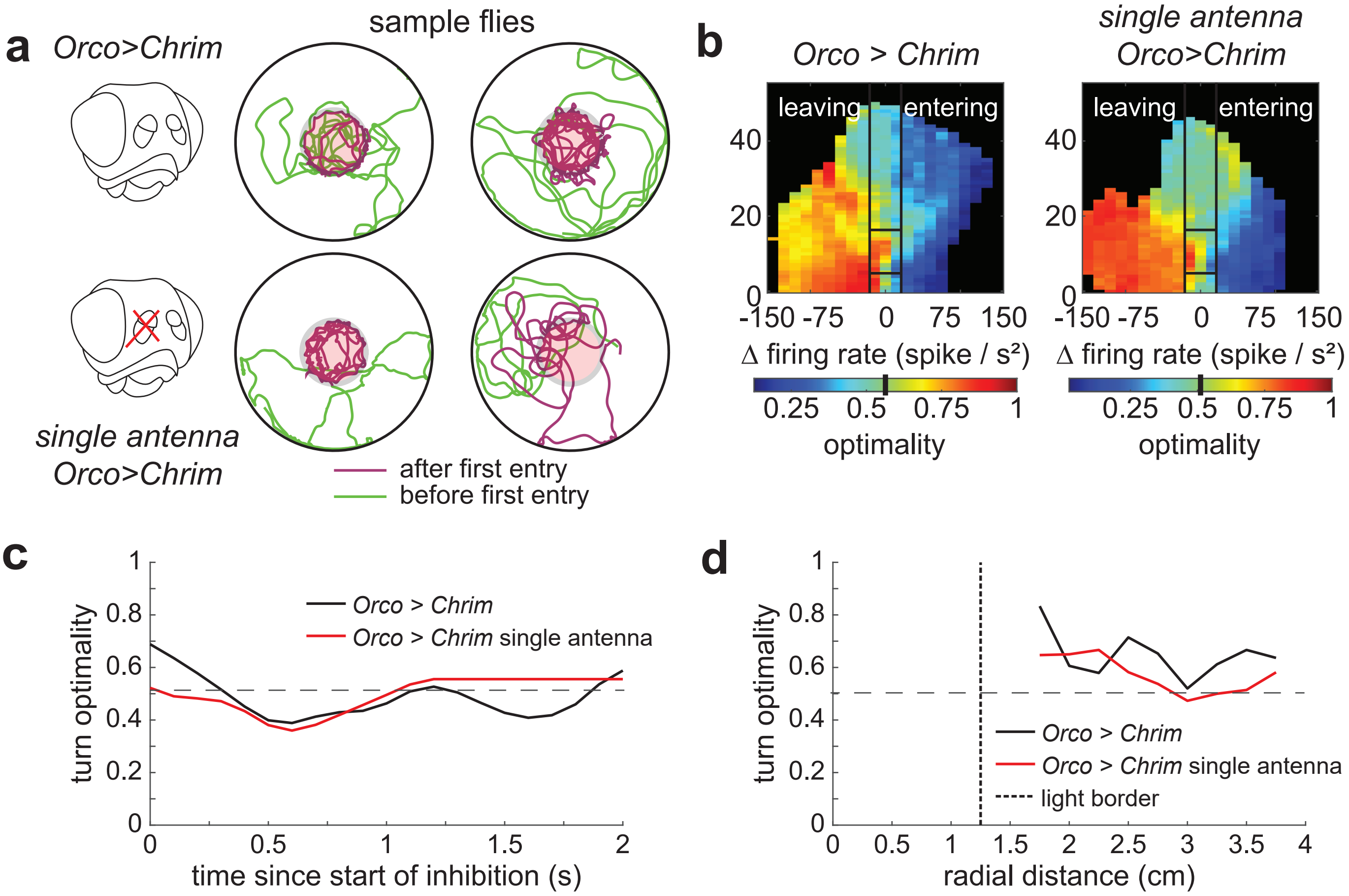

Figure S19. Directed odor tracking can not be explained purely from comparisons between antennae. a. Sample trajectories of *Orco* > *Chrimson* flies (top) and *Orco* > *Chrimson* flies with the right antenna removed (bottom) shows that while some some flies can still make tight border weaving movements, others make larger loops. **b.** Turn optimality (defined as the percentage of optimal turns) for *Orco* > *Chrimson* and single antenna *Orco* > *Chrimson* relative to baseline. Black lines separate out the sensorimotor mapping into 5 broad regions based on firing rate and change in firing rate. Baseline turn optimality is shown as a black bar in the colorbar. **c.** Turn optimality when ORN activity is inhibited as a function of time since the start of the inhibition period. **d.** Turn optimality when ORN activity returns to baseline firing rate as a function of radial location away from the center of the arena shows that *Orco* > *Chrimson* flies do have a slightly higher than chance (0.5) turn optimality irrespective of distance from the stimulus zone.

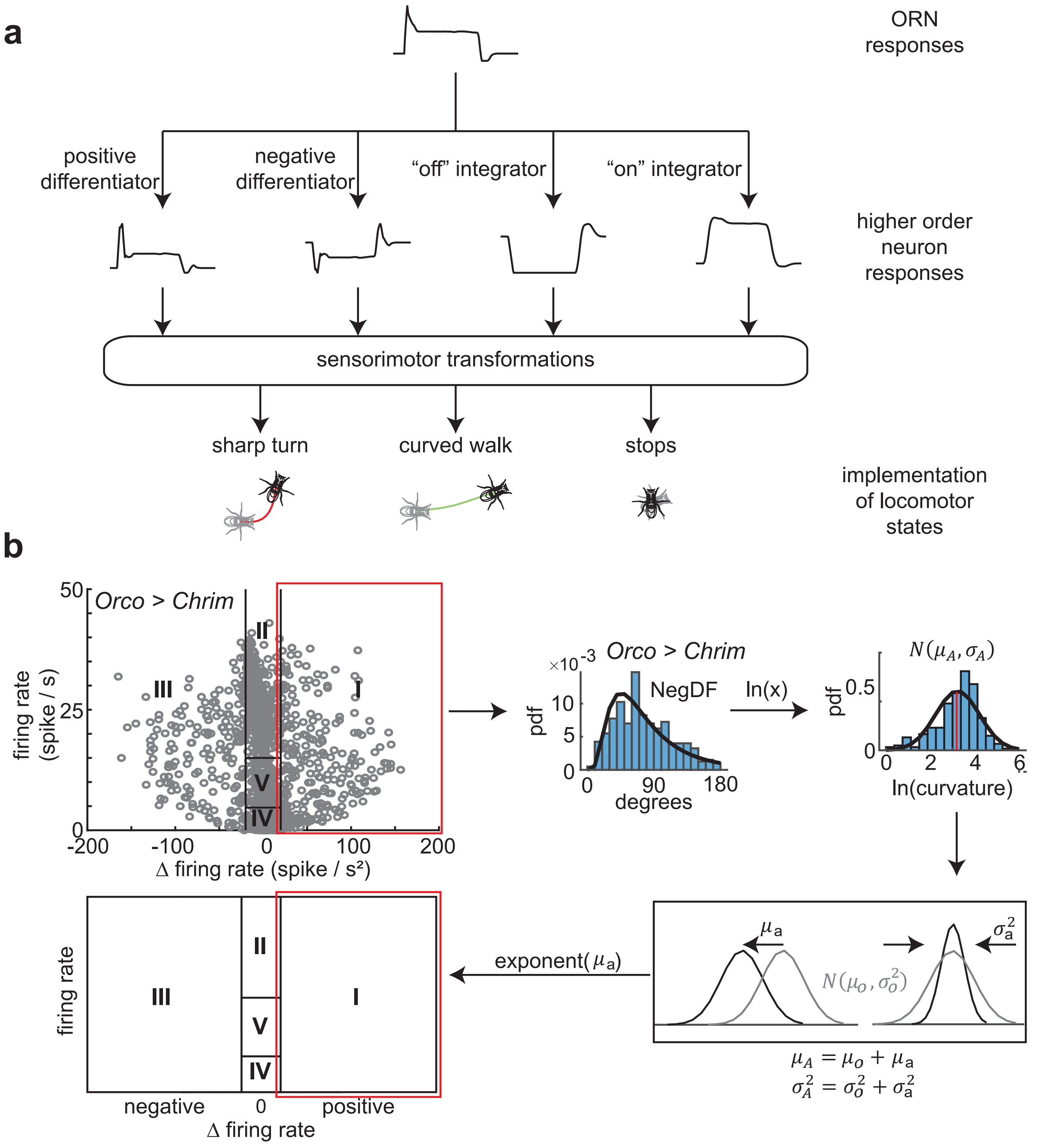

Figure S20. Modeling the effect of a single class (or group) of ORN activity on locomotor kinematics. **a.** Signals from each ORN is carried through parallel channels through higher order neurons that act as positive and negative differentiators and integrators of ORN activity. These channels lead to downstream sensorimotor circuits that modulates the kinematics of different locomotor states. We only show 4 channels for convienience. There can be many more. Our analysis is based on 5 regions. **b.** Locomotor trajectories can be classified into 5 broad regions based on firing rate and change in firing rate. The distribution of locomotor kinematics (sharp turn curvature for *Orco>Chrim* is shown) follows a gaussian distribution after taking a natural log transform. The effect of ORN activity is modeled by the shift in the mean of this distribution as compared to baseline (the distribution prior to first entry) and either increasing or decreasing the variance in the distribution as compared to baseline. The mean shift transformed back into the kinematics space can then be summarized in a schematic reflecting each of the five broad ORN activity regions.

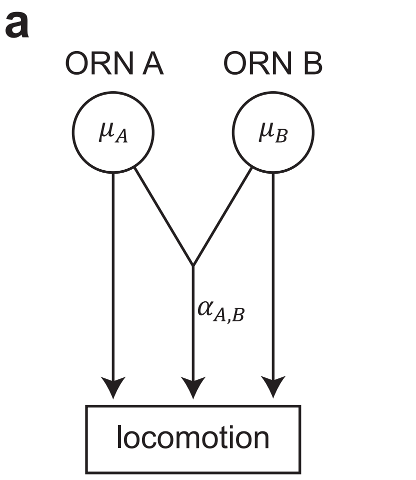

$$\mu_{AB} = \mu_O + \mu_A + \mu_B + \alpha_{A,B}\mu_A\mu_B$$
$$\sigma_{AB}^2 = \sigma_O^2 + \sigma_A^2 + \sigma_B^2 + 2\sigma_{A,B}$$

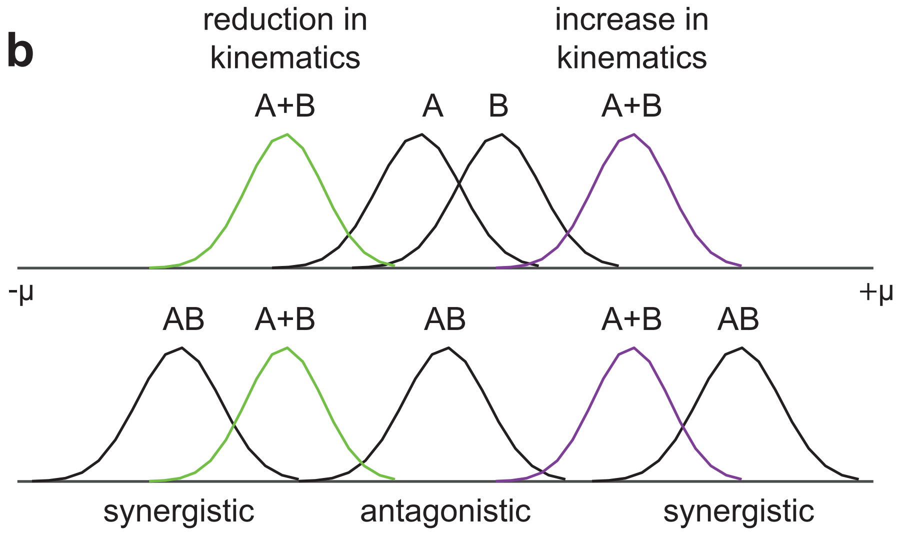

μ_A	μ_B	$\alpha_{A,B}$	
+	+	+	synergistic
+	+	-	antagonistic
+	-	+/-	N/A
-	+	+/-	N/A
-	-	+	antagonistic
-	-	-	synergistic

Figure S21. Rules of combination for two sets of ORNs. a. Schematic of individual ORN effect on locomotion and an combinatorial effect on locomotion. **b (top).** Under separate pathways, the net effect on locomotion when coactivating two sets of ORNs is the sum of each set of ORN. ORNs interact synergistically when ORNs that cause an increase in locomotor kinematics shows an higher increase in kinematics when co-activated than the sum of the individual effects. The reverse is true when ORNs decrease the locomotor kinematics. Meanwhile, if the net effect on locomotion is less than the sum of it's parts, then the effect is antagonistic. **b (bottom).** Whether any 2 sets of ORNs interact in a synergistic or antagonistic manner is dependent on the sign of the contribution of each individual set of ORN and the interaction between them. Scenarios where the effect is classified as N/A can be further delineated as being ORN A dominant, ORN B dominant, linear summation or other unclassified types (see methods for details).

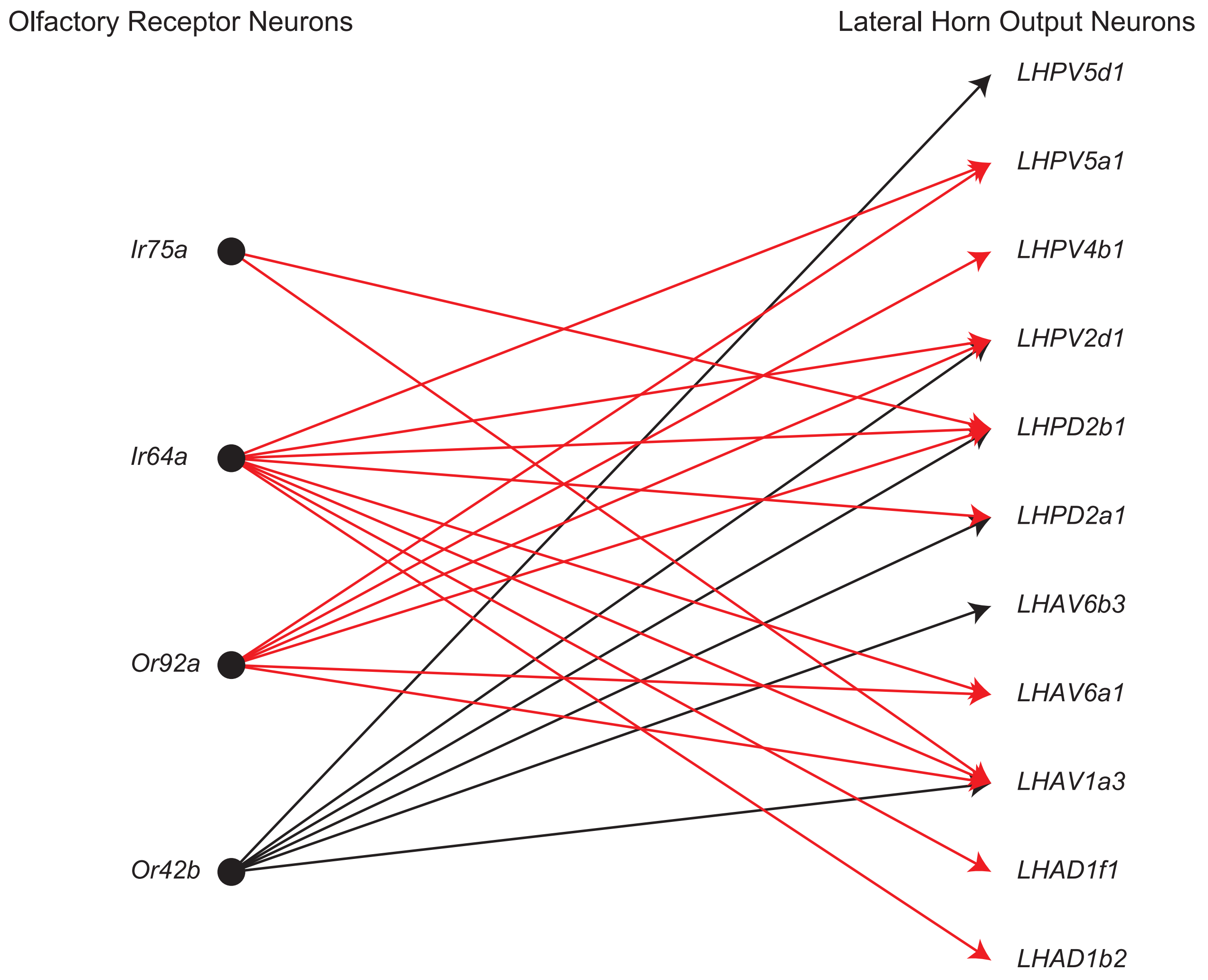

Figure S22. A small sample from connectomics data show convergent and divergent integration of olfactory information in lateral horn output neurons (LHON). These circuit motifs might underlie the rules of integration we have described. Each arrow indicates that a direct connection (via uniglomerular projection neurons) exists between an ORN class used in this study and the respective LHON class. This represents a small fraction of the actual connections downstream of the antennal lobe and illustrates 1) how the divergence of information from each ORN class support differential effects of the ORN on motor parameters, 2) that there can be diverse rules of integration that depend on ORN-type and motor parameter.

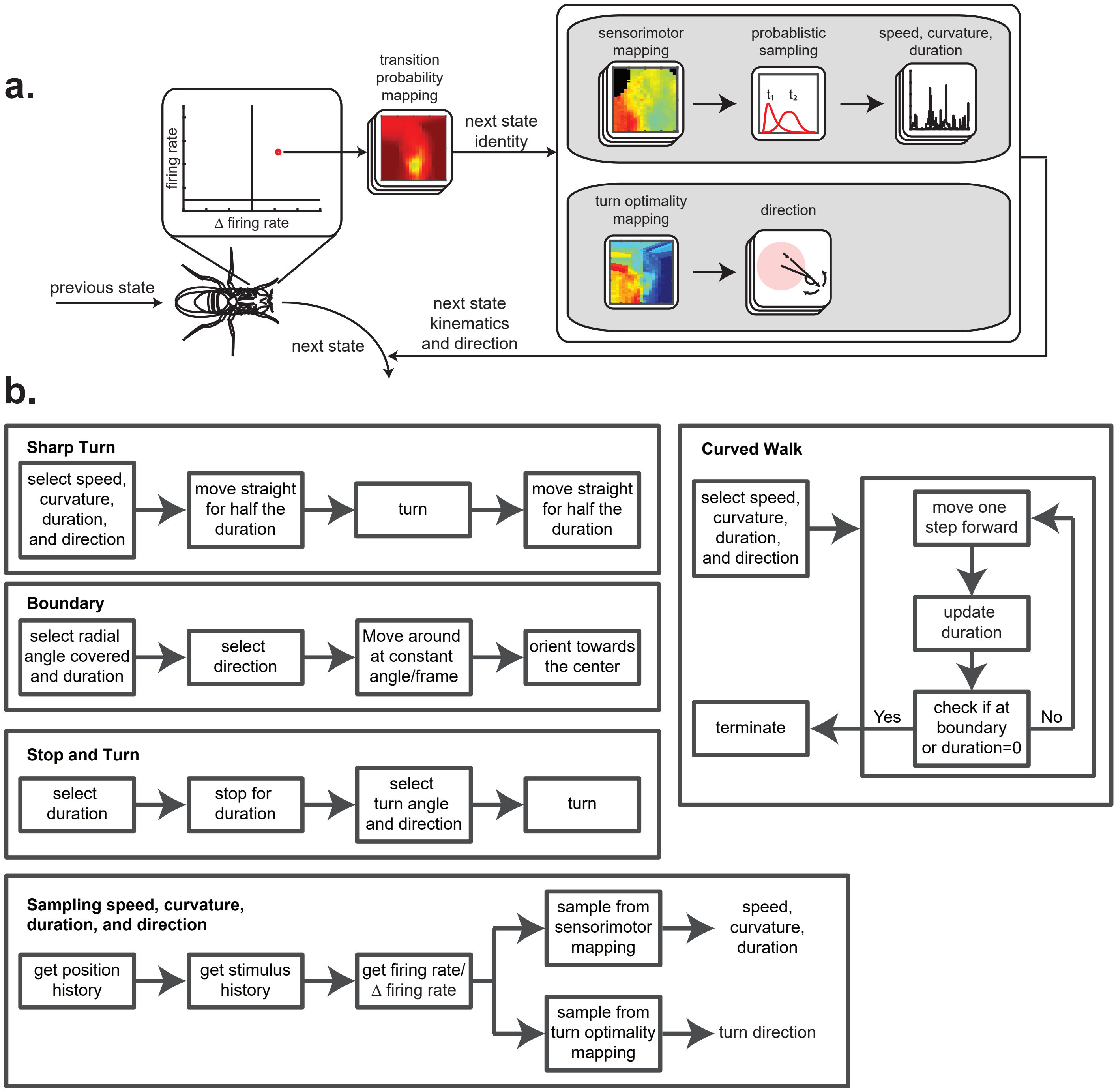

Figure S23. Details of the agent based model. a. Synthetic flies can be in one of four states: sharp turn, curved walk, boundary, and stops. During state transitions, flies experience some ORN firing rate and change in firing rate activity. They will transition to a new state based on the ORN activity. Based on the ORN activity and time since first stimulus encounter, they will sample from an lognormal distribution for the new state's speed, curvature, and duration. Synthetic flies will also choose a direction based on the turn optimality mapping based on the ORN activity and time since first stimulus. **b.** Pipeline of steps synthetic flies take during each of the four states. Like directed runs, a sharp turn will transition into a boundary state if the fly reaches the boundary prior to the end of the sharp turn instance. The fly leaves the boundary by orienting towards the center of the arena at an angular offset of +/- 10 degrees. Adapted from "Mechanisms underlying attraction to odors in walking Drosophila." by Tao, L., Ozarkar, S., & Bhandawat, V. 2020, PLOS Computational Biology, 16(3), e1007718.

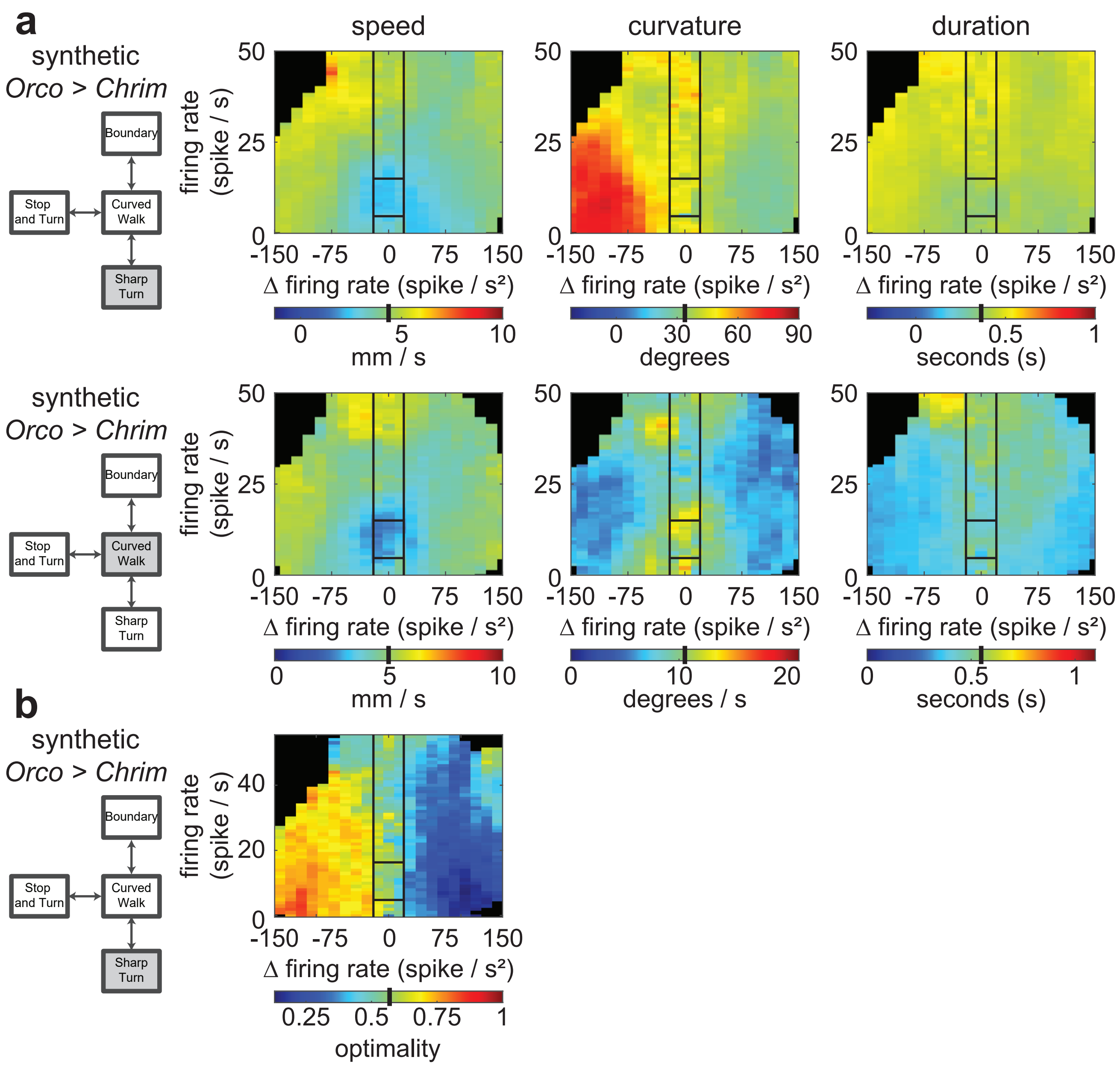

Figure S24. An agent based model of flies based on locomotor kinematics and turn optimality can preserves the sensorimotor mappings of empirical flies. a. Sensorimotor mapping of the effect of ORN activity on Sharp Turn (**top**) and Curved Walk (**bottom**) speed, curvature, and duration for synthetic flies. **b.** Turn optimality (defined as the percentage of optimal turns) for synthetic flies relative to baseline. Black lines separate out each sensorimotor mapping into 5 broad regions based on firing rate and change in firing rate. Baseline turn optimality is shown as a black bar in the colormap.

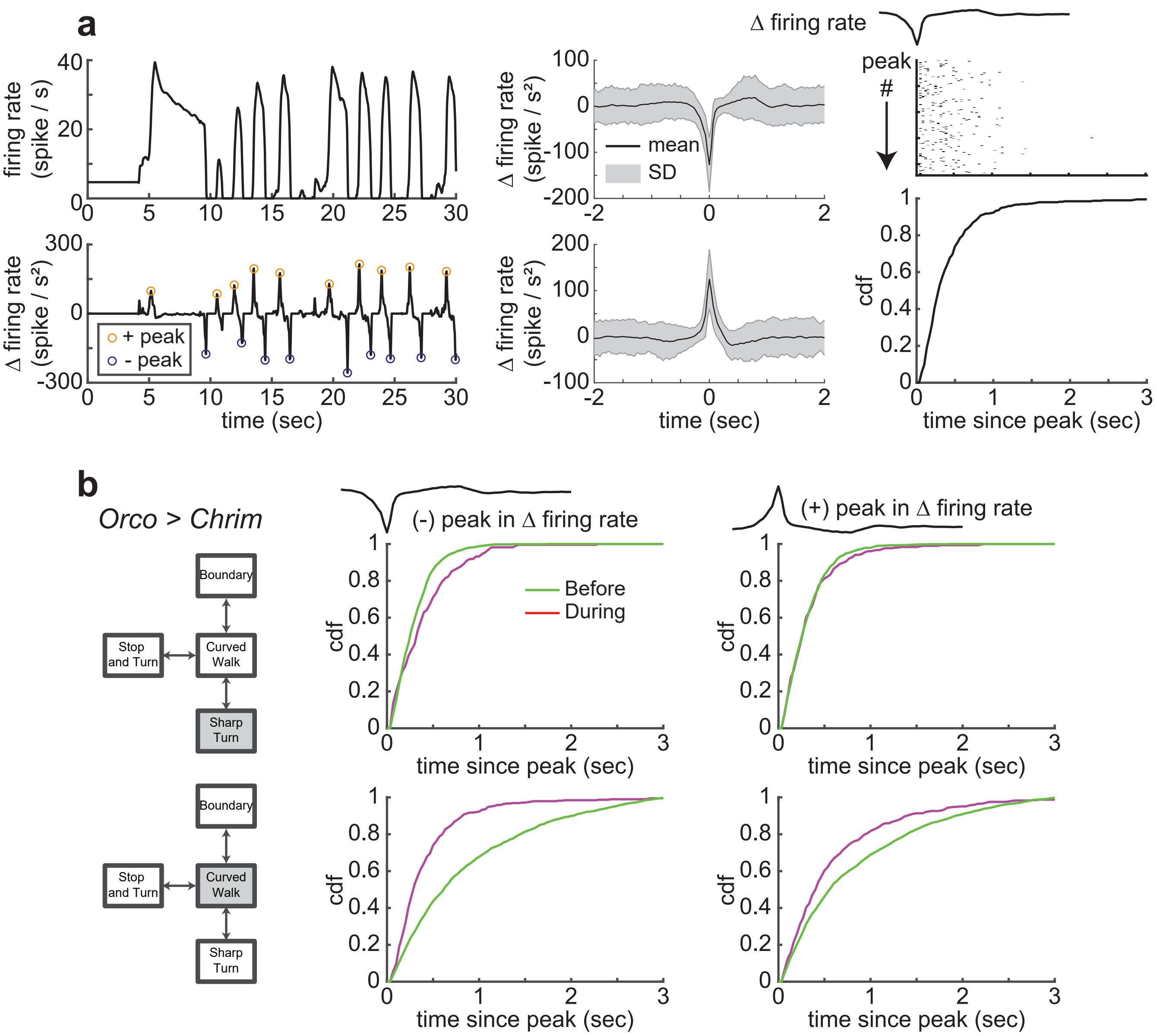

Figure S25. Large change in ORN firing rate causes flies to transition from a curved walk to sharp turn. a. Left: Sample 30 second firing rate and change in firing rate of an Orco>Chrimson fly. Circle show positive (orange) and negative (blue) peaks in the change in firing rate >50spikes/s^2 and <-50 spikes/s^2 respectively. **Center:** Mean and standard deviation of positive and negative peaks aligned by time of peak in change in firing rate. **Right:** Raster of time after negative peak in change in firing rate when the first transition out of a curved walk state occurs. Each row represents an instance where the fly experiences a negative peak in change in firing rate when in the curved walk state. These transition times are summarized using the cumulative distribution function (cdf). **b.** CDF for sharp turns (top row) and curved walks (bottom row) time to state transition since negative (middle column) and positive (right column) peak in change in firing rate. Before CDFs are constructed by randomly sampling time points to serve as "peaks in change in firing rate" that belong to curved walk and sharp turn tracks before first entry.

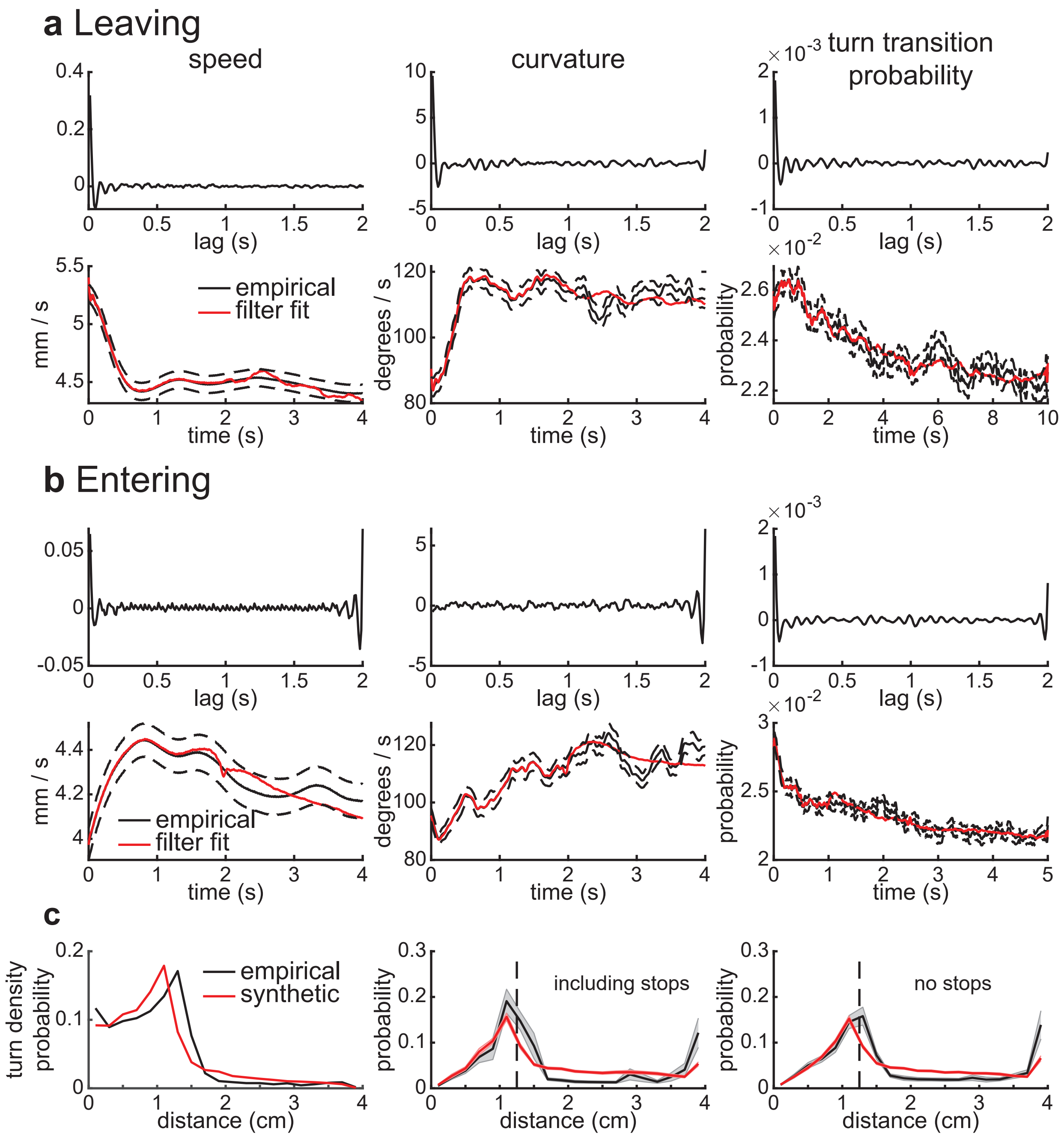

Figure S26. A model that incorporates linear filter to model behavior at the light border and KNN elsewhere matches the fly behavior well. a. Top: Linear filter transformation from firing rate to speed, curvature, and probability of transition into a sharp turn when flies are leaving the light zone. Bottom: speed, curvature, and probability of sharp turn transition probability after flies leave. Error bars show standard deviation of resampled trajectories. Leaving tracks are aligned and defined as a trough in the change in firing rate less than -15 spikes/s^2. **b.** Same as **a**, but for when flies are entering the light arena. Entering tracks are defined by a change in firing rate greater than 15 spikes/s^2. **c.** Turn density (left) and spatial distribution (middle) of the empirical and synthetic flies after the stimulus is turned on. Right: The small discrepency in the peak in spatial distribution is largely due to empirical flies stopping just inside the light zone. Radial occupancy shows mean +/- SEM.

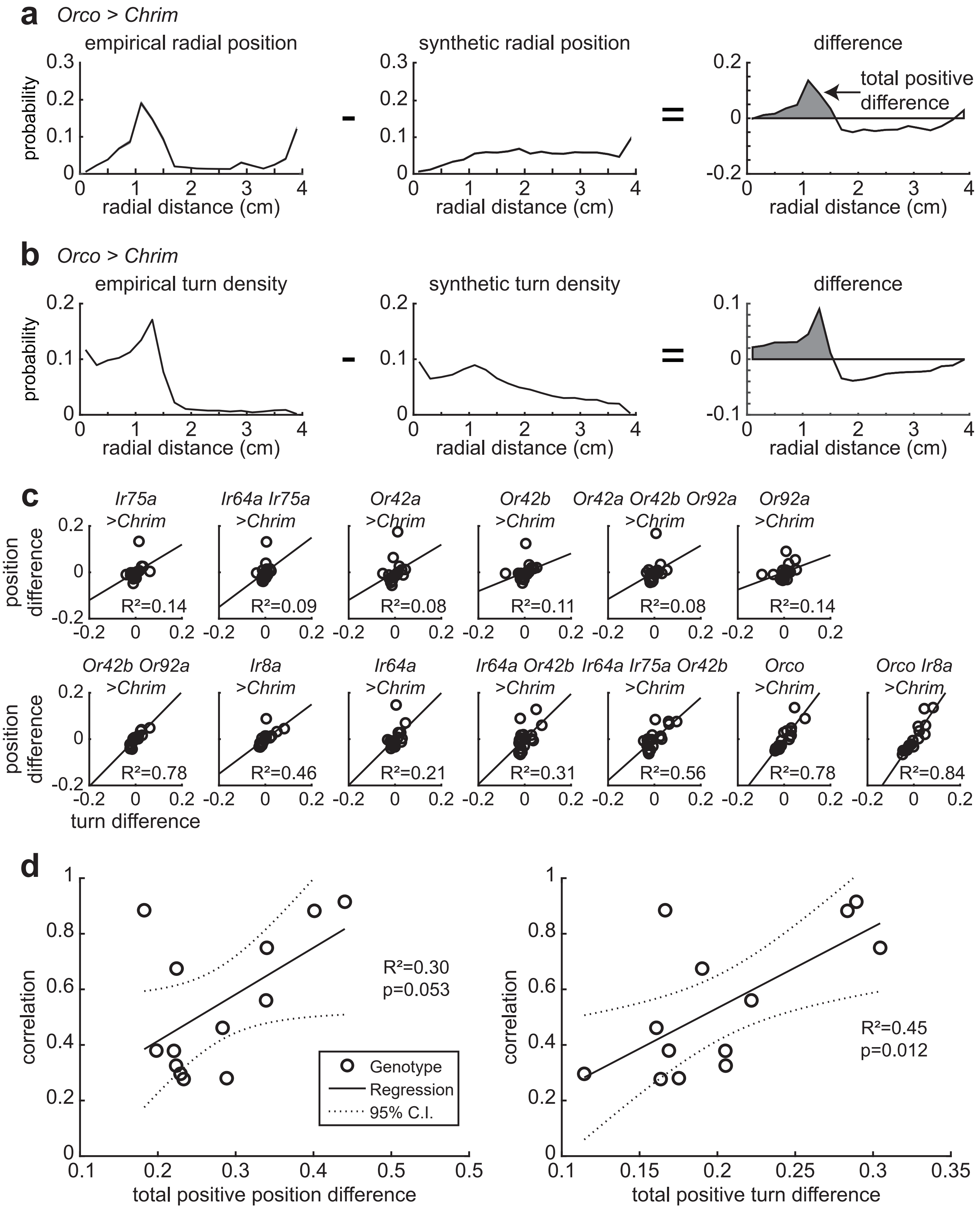

Figure S27. Radial occupancy differences between empirical and synthetic flies is correlated with turn density differences. a. Difference in radial occupancy between *Orco > Chrimson* empirical flies (left) and synthetic flies (middle). The total positive difference is calculated represents the amount of change between the empirical and synthetic. **b.** Same as **a**, but for turn density. **c.** Scatter plots of the difference in turn density against difference in radial occupancy for each genotype listed in the same order as the main Figure 7 shows a strong correlation between the time. The correlation becomes larger as the difference between empirical and synthetic is larger. **d.** The difference in radial occupancy is more correlated with the difference in turn density for genotypes where there is a large difference in either radial occupancy (left) or turn density (right) between the empirical and synthetic.
