# Supplementary material for: Sensorimotor transformation underlying odor-modulated locomotion in walking Drosophila
Source: Nat Commun. 2023 Oct 26;14:6818. doi: 10.1038/s41467-023-42613-8 (PMC10603174; doi:10.1038/s41467-023-42613-8)
Supplement: Supplementary file 3 — Reporting Summary [file 41467_2023_42613_MOESM3_ESM.pdf]

nature portfolio

Corresponding author(s): Vikas Bhandawat

Last updated by author(s): Jun 26, 2023

Reporting Summary

Nature Portfolio wishes to improve the reproducibility of the work that we publish. This form provides structure for consistency and transparency in reporting. For further information on Nature Portfolio policies, see our Editorial Policies and the Editorial Policy Checklist.

Statistics

For all statistical analyses, confirm that the following items are present in the figure legend, table legend, main text, or Methods section.

n/a	Confirmed	
☐	☒	The exact sample size (*n*) for each experimental group/condition, given as a discrete number and unit of measurement
☒	☐	A statement on whether measurements were taken from distinct samples or whether the same sample was measured repeatedly
☐	☒	The statistical test(s) used AND whether they are one- or two-sided<br>*Only common tests should be described solely by name; describe more complex techniques in the Methods section.*
☐	☒	A description of all covariates tested
☐	☒	A description of any assumptions or corrections, such as tests of normality and adjustment for multiple comparisons
☐	☒	A full description of the statistical parameters including central tendency (e.g. means) or other basic estimates (e.g. regression coefficient) AND variation (e.g. standard deviation) or associated estimates of uncertainty (e.g. confidence intervals)
☐	☒	For null hypothesis testing, the test statistic (e.g. *F*, *t*, *r*) with confidence intervals, effect sizes, degrees of freedom and *P* value noted<br>*Give P values as exact values whenever suitable.*
☒	☐	For Bayesian analysis, information on the choice of priors and Markov chain Monte Carlo settings
☐	☒	For hierarchical and complex designs, identification of the appropriate level for tests and full reporting of outcomes
☒	☐	Estimates of effect sizes (e.g. Cohen's *d*, Pearson's *r*), indicating how they were calculated

Our web collection on statistics for biologists contains articles on many of the points above.

Software and code

Policy information about availability of computer code

Data collection	All data was collected through custom software written in MATLAB2019B; also tested on MATLAB 2022B
Data analysis	All analysis was performed using custom code written in MATLAB2019B; also tested on MATLAB 2022B (DOI: 10.5281/zenodo.8190933).<br><br>Apart from the code release above, the following code from previous work (Tao, Ozarkar, Bhandawat) was used:<br>Optogenetics arena fly tracker https://github.com/bhandawatlab/CircularArenaTrackingCode<br><br>Delineation of movement states (DrosoRT) Tao, Ozarkar, Bhandawat 2020 https://github.com/bhandawatlab/DrosoRT<br><br>Single sensillum recording and spike sorting GUI https://github.com/bhandawatlab/Single-Sensillum-Spike-Sorting-GUI<br><br>Optogenetics arena fly tracker Tao, Ozarkar, Bhandawat 2020 https://github.com/bhandawatlab/CircularArenaTrackingCode<br><br>Delineation of movement states (DrosoRT) Tao, Ozarkar, Bhandawat 2020 https://github.com/bhandawatlab/DrosoRT<br><br>Single sensillum recording and spike sorting GUI https://github.com/bhandawatlab/Single-Sensillum-Spike-Sorting-GUI<br><br>any2ufmf (part of The Caltech Multiple Walking Fly Tracker) Branson et al., 2009 http://ctrax.sourceforge.net/any2ufmf.html

For manuscripts utilizing custom algorithms or software that are central to the research but not yet described in published literature, software must be made available to editors and reviewers. We strongly encourage code deposition in a community repository (e.g. GitHub). See the Nature Portfolio guidelines for submitting code & software for further information.

Data

Policy information about availability of data

All manuscripts must include a data availability statement. This statement should provide the following information, where applicable:

- Accession codes, unique identifiers, or web links for publicly available datasets
- A description of any restrictions on data availability
- For clinical datasets or third party data, please ensure that the statement adheres to our policy

https://doi.org/10.6084/m9.figshare.22776428

Research involving human participants, their data, or biological material

Policy information about studies with human participants or human data. See also policy information about sex, gender (identity/presentation), and sexual orientation and race, ethnicity and racism.

Reporting on sex and gender	NA
Reporting on race, ethnicity, or other socially relevant groupings	NA
Population characteristics	NA
Recruitment	NA
Ethics oversight	NA

Note that full information on the approval of the study protocol must also be provided in the manuscript.

Field-specific reporting

Please select the one below that is the best fit for your research. If you are not sure, read the appropriate sections before making your selection.

☒ Life sciences ☐ Behavioural & social sciences ☐ Ecological, evolutionary & environmental sciences

For a reference copy of the document with all sections, see nature.com/documents/nr-reporting-summary-flat.pdf

Life sciences study design

All studies must disclose on these points even when the disclosure is negative.

Sample size	Sample sizes were based on previous work starting with https://doi.org/10.7554/eLife.11092.
Data exclusions	No data were excluded
Replication	NA. The study design compares the behavior of flies of different genotypes. The conclusions are based on the distribution of behavior of flies of a given genotype as no two flies are exactly the same.
Randomization	NA. All experiments were done on flies with identical genetic background reared in exactly the same way. The main comparison is between flies of the same genotype that are either fed retinal or not fed retinal. Half the flies from each vial are fed retinal; there is no selection regarding which flies are fed retinal; in this sense, the experimental group and control group are selected at random. But, this is fundamentally different from how people are assigned to random groups in a clinically trial because in that case subjects have vastly different genetics and life-style. Similarly, which flies (experimental or control) were used on a given day were chosen at random.
Blinding	NA. There is no human involvement in analysis. All data is analyzed automatically

Reporting for specific materials, systems and methods

We require information from authors about some types of materials, experimental systems and methods used in many studies. Here, indicate whether each material, system or method listed is relevant to your study. If you are not sure if a list item applies to your research, read the appropriate section before selecting a response.

Materials & experimental systems

n/a	Involved in the study
☒	☐ Antibodies
☒	☐ Eukaryotic cell lines
☒	☐ Palaeontology and archaeology
☐	☒ Animals and other organisms
☒	☐ Clinical data
☒	☐ Dual use research of concern
☒	☐ Plants

Methods

n/a	Involved in the study
☒	☐ ChIP-seq
☒	☐ Flow cytometry
☒	☐ MRI-based neuroimaging

Animals and other research organisms

Policy information about studies involving animals; ARRIVE guidelines recommended for reporting animal research, and Sex and Gender in Research

Laboratory animals	Drosophila melanogaster. The flies were 3-5 days old. Multiple genotypes were used and are listed in methods.
Wild animals	No wild animals were used
Reporting on sex	All Drosophila melanogaster olfaction experiments are done on females.
Field-collected samples	No field samples were collected.
Ethics oversight	No study protocols are required for invertebrate work. That is no ethical approval or guidance was required as the study subjects are invertebrates/

Note that full information on the approval of the study protocol must also be provided in the manuscript.
